# Adiabatic Shear Localization in Metallic Materials: Review

**DOI:** 10.3390/ma17215365

**Published:** 2024-11-01

**Authors:** Xinran Guan, Shoujiang Qu, Hao Wang, Guojian Cao, Aihan Feng, Daolun Chen

**Affiliations:** 1 Shanghai Key Laboratory of D&A for Metal-Functional Materials, School of Materials Science & Engineering, Tongji University, Shanghai 201804, China; 13101618335@sina.cn (X.G.); aihanfeng@tongji.edu.cn (A.F.); 2 Institute of Metal Research, Chinese Academy of Sciences, Shenyang 110016, China; haowang@imr.ac.cn; 3 Key Laboratory for Light-Weight Materials, Nanjing Tech University, Nanjing 210009, China; caoguojian@njtech.edu.cn; 4 Department of Mechanical, Industrial and Mechatronics Engineering, Toronto Metropolitan University, Toronto, ON M5B 2K3, Canada

**Keywords:** adiabatic shear instability, adiabatic shear band, materials under extreme conditions, dynamic behavior

## Abstract

In advanced engineering applications, there has been an increasing demand for the service performance of materials under high-strain-rate conditions where a key phenomenon of adiabatic shear instability is inevitably involved. The presence of adiabatic shear instability is typically associated with large shear strains, high strain rates, and elevated temperatures. Significant plastic deformation that concentrates within a adiabatic shear band (ASB) often results in catastrophic failure, and it is necessary to avoid the occurrence of such a phenomenon in most areas. However, in certain areas, such as high-speed machining and self-sharpening projectile penetration, this phenomenon can be exploited. The thermal softening effect and microstructural softening effect are widely recognized as the foundational theories for the formation of ASB. Thus, elucidating various complex deformation mechanisms under thermomechanical coupling along with changes in temperatures in the shear instability process has become a focal point of research. This review highlights these two important aspects and examines the development of relevant theories and experimental results, identifying key challenges faced in this field of study. Furthermore, advancements in modern experimental characterization and computational technologies, which lead to a deeper understanding of the adiabatic shear instability phenomenon, have also been summarized.

## 1. Overview of Adiabatic Shear Instability

**Figure 1:**
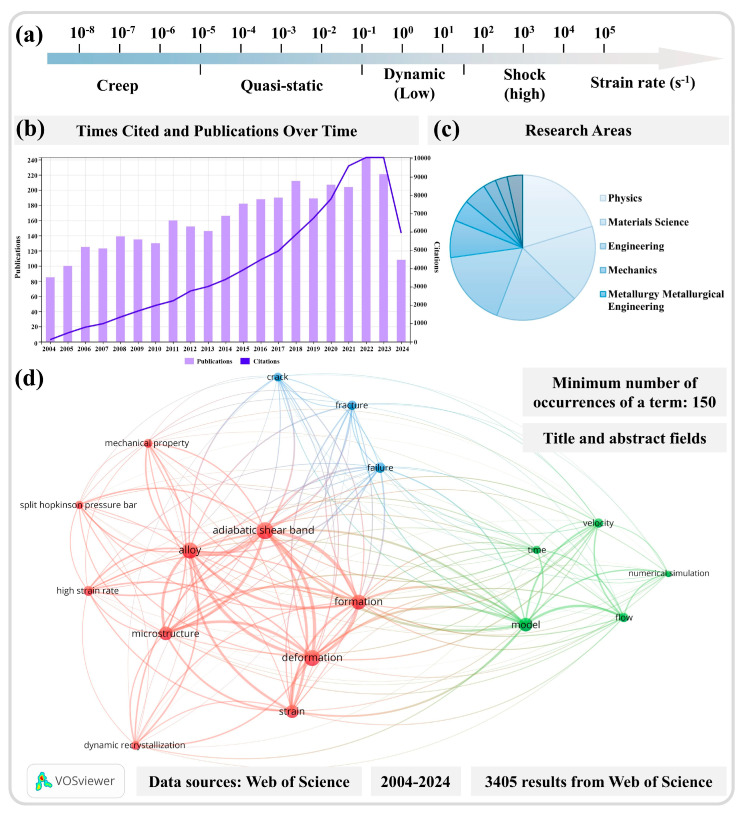
(**a**) Classification of mechanical testing based on the strain rate [7]. (**b**) The number of articles published annually on the topic of adiabatic shear instability over the past 20 years and the corresponding citation counts. (**c**) Research areas of articles published in the last 20 years. (**d**) Analysis of the relevance of literature on adiabatic shear instability retrieval results (data sourced from the Web of Science database). Reproduced with permission from Elsevier and open-access websites.

Localization of plastic deformation in metallic materials occurs in various alloys and under different loading conditions. This includes strain localization during fatigue due to the formation of irreversible slip bands [1], shear bands within the material at low strain rates, and the necking phenomenon during tensile processes, as well as adiabatic shear bands (ASBs) under high-strain-rate conditions. The emergence of these heterogeneous deformation phenomena is often manifested as a decline in the stress–strain curve, accompanied by the initiation and propagation of cracks, ultimately leading to fracture [2]. Thus, plastic deformation localization is of vital importance for the application and mechanical behavior of metallic materials [3]. The preceding discussion highlights the widespread presence of plastic localization phenomena in metallic materials, which typically occurs prior to ductile fracture [4]. Furthermore, the mechanisms underlying plastic deformation localization under different strain rates exhibit variations that are closely related to the selection of deformation mechanisms in metallic materials at varying strain rates [5]. Generally, the phenomenon of adiabatic shear instability occurs under high-speed impact conditions at strain rates exceeding 10^3^ s^−1^ [6] as defined by different loading strain rates, as illustrated in Figure 1a [7,8].

As a pervasive phenomenon of plastic instability in service under high-strain-rate conditions [8,9,10,11,12], the high strain rate allows only an extremely short deformation time on a microsecond scale. Consequently, it is generally assumed that there is negligible thermal interaction between the material and its outer environment, which is referred to as adiabatic conditions. Additionally, due to the highly localized nature of the deformation, narrow bands with a width of approximately 5 to 100 μm are formed within the material, known as ASBs [13,14]. In 1878, Tresca [15] discovered that significant heat could be generated within metals during the forging process, even to the extent that metals could reach a dark red color. He also observed marked thermal effects during the forging of iridium–platinum alloys, where a glowing region resembling the letter ‘x’ formed, marking the first recorded detection of ASBs within materials. Since this discovery, the phenomenon has been extensively studied. In 1944, Zener and Hollomon [16] conducted mechanical tests across a wide range of temperatures (from room temperature to liquid nitrogen temperature) and strain rates (ranging from 10^−5^ to 10^3^ s^−1^, covering quasi-static to impact conditions) to investigate how the stress–strain relationship of steel is affected by adiabatic effects under conditions of high-rate deformation. Their principal findings indicated that higher strain rates resulted in a transition of deformation from isothermal to adiabatic conditions, where the adverse effect of temperature rise on stress outweighed the beneficial effect of strain hardening, leading to a negative slope in the stress–strain curve. This also implied an intrinsic instability present in the material during deformation; local regions experienced further weakening due to increased deformation, i.e., concentrating the deformation in these areas leads to what is termed the “thermoplastic instability theory”. This theory has guided nearly a century of research in this field, and up to now, researchers continue to employ it for analyzing and interpreting the obtained results while correctly defining the formation of ASBs as induced by plastic instability [17]. It is worth noting that both the initial discovery of ASBs and temperature rise, as key parameters in thermoplastic theories, hold a significant impact. Given that adiabatic shear instability occurs over very small temporal and spatial scales, predicting and measuring it in practice remains a challenging endeavor. Estimations of temperature in the adiabatic shear zones, along with microstructural analyses, indirectly suggest that the temperature may have reached levels conducive to DRX [18,19], phase transitions [20,21], and even in certain materials, melting temperatures [22,23]. With the continuous advancement in instrumentation science, the high-speed measurement and capture of temperatures in the adiabatic shear instability process have become feasible; however, there remains a discrepancy between existing actual measurement results and theoretically estimated or inferred temperatures. This discrepancy includes variations in temperature levels and poses challenges regarding the stages of temperature elevation in the adiabatic shear instability process [24,25]. As one of the most critical factors influencing the deformation of metallic materials, the temperature of the adiabatic shear region is crucial for the development of adiabatic shear instability and the microstructural evolution of plastic localization regions. A more detailed discussion and analysis of this topic will be conducted subsequently.

To better analyze and discuss the phenomenon of localized plastic deformation under high-strain-rate conditions, the review will be divided into the following five sections:

**Overview of adiabatic shear instability:** This section includes a relevant overview of theories such as the “thermoplastic instability theory” and introduces potential fields where adiabatic shear phenomena may occur, thereby illustrating the necessity of investigating adiabatic shear behavior. By summarizing the adiabatic shear theory and conducting the search and analysis of literature, we can determine the general research directions within the field and clarify two primary routes of discussion for this review.

**Research methods on adiabatic shear instability:** This part summarizes the advantages and limitations of various loading methods and their applicable ranges, as well as the samples of different geometries used in the study of adiabatic shear deformation. Since different experimental methods can influence the subsequent microstructural characterization analysis, temperature estimation, and actual measurement of the adiabatic shear zone, an overview of the research methods is necessary and will serve as a foundation for further discussion and analysis.

**Various deformation mechanisms within ASB:** This section discusses the formation mechanisms of the ASB, summarizes the multiple deformation mechanisms that occur within the adiabatic shear region, and analyzes their intrinsic mechanisms. The discussion on microstructures precedes the discussion on temperatures because research on the evolution of microstructures in adiabatic shear zones is closely related to temperatures, which subsequently leads to a final focused discussion on temperatures.

**Temperature evolution in the adiabatic shear region:** As a thermomechanical coupling phenomenon under extreme deformation conditions, the temperature evolution in the adiabatic shear region is crucial for the emergence of its internal deformation mechanisms and the development of adiabatic shear localization. Hence, it will be analyzed and summarized.

**Concluding remarks and prospects:** This final section will summarize the current research findings and anticipate future research directions.

To provide a more thorough and comprehensive review of this research topic, a literature retrieval analysis of existing research results was first conducted. By searching the Web of Science database using the terms “adiabatic shear band”, “adiabatic shear localization”, “adiabatic shear instability”, and “adiabatic shear”, we analyzed the research outcomes in the field of adiabatic shear localization over the past two decades, including the annual publication and citation counts as well as the related fields of research, as illustrated in Figure 1b,c. Over the last 20 years, more than 100 papers on this topic have been published annually, showing a gradual increasing trend, with approximately 200 papers being published each year in the past six years. Furthermore, the citation count from the Web of Science has steadily increased over the past two decades, correlating with the steadily increasing publications of research findings each year. This indicates that this research area continues to receive considerable and widespread attention from numerous scholars. Of the over 3000 papers published on this topic, the majority can be attributed to research in fields such as physics, materials science, engineering technology, mechanics, and metallurgical engineering, with physics and materials science making up the largest proportions.

A further analysis of the searched results related to the theme of adiabatic shear instability is performed, where the terminology appearing in the titles and abstracts has been screened and refined. A minimum occurrence of 150 repetitions of terms was set from the 3405 retrieved results to conduct the relevance analysis. In Figure 1d, the size of solid circles represents the frequency of term repetition, with a larger circle representing a higher occurrence rate. The lines connecting different circles represent correlations in research, while different colors reflect an approximate classification of this research field derived from the term analysis. It was observed that the research on adiabatic shear instability over the past 20 years can be broadly categorized into three main classifications. The studies on the microstructures of ASBs belong to one of the highest proportions, which are heavily related to the formation and deformation mechanisms of ASBs. In studies concerning microstructures, dynamic recrystallization (DRX) also represents a significant portion. The high frequency of the term “split Hopkinson pressure bar” indicates the widespread adoption of this experimental method. Other primary categories encompass modeling and numerical simulation studies addressing adiabatic shear instability, followed by studies on material fracture and failure, which are inevitably related to adiabatic shear instability. The literature review of nearly 20 years of research reveals an extensive volume of literature on this topic. Although this review aims to systematically discuss and analyze this research field, the number of references cited in this review remains quite limited compared to the entirety of the field.

**Figure 2:**
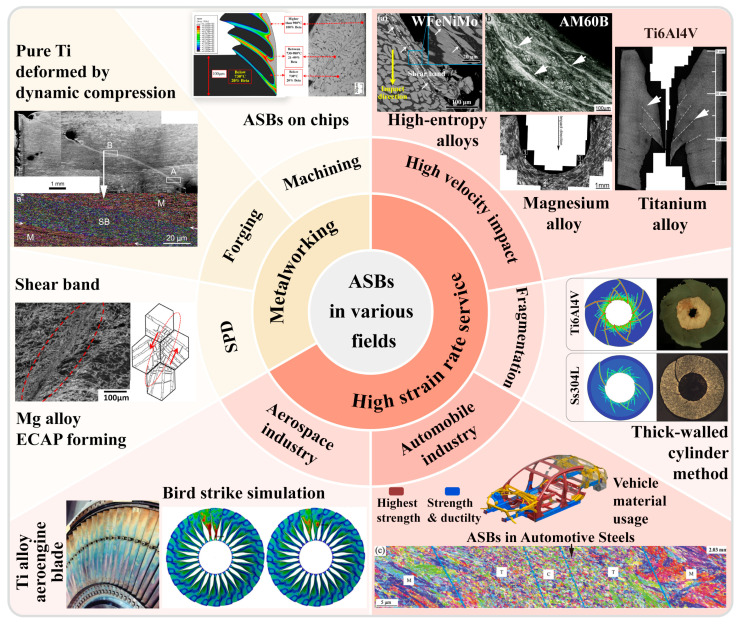
Illustration of adiabatic shear instability prevalent in various fields of metalworking processes such as cutting [55], forging [39], and large plastic deformation [41], and high-strain-rate service conditions such as high-velocity impact [30,31,34,56], aerospace [57,58] and automotive industries [52,54]. Reproduced with permission from Elsevier and open-access websites.

The widespread occurrence of plastic instability phenomena and the increasingly stringent requirements for further applications of materials have led to this becoming a long-standing concern in the field [26]. The high-velocity impact applications have shown significant interest in the adiabatic shear instability phenomenon, as illustrated in Figure 2. In early studies, the adiabatic shear localization phenomenon generated during projectile penetration was deemed detrimental, as this instability could facilitate deformation or even fracturing of the projectile, thereby undermining its penetration capability [27,28]. However, certain “self-sharpening” projectile materials have leveraged this instability to enhance their penetrating power [29]. The tungsten-based high entropy alloy self-sharpening penetrator material, depicted in Figure 2, induces DRX through the significant soft-hard phase disparity within the material. This softening results in reduced localized stresses, promoting the formation of ASBs [30]. Additionally, the formation of these shear bands at high strain rates at the projectile tip edges causes certain deformed portions to shed, maintaining a sharp projectile shape and improving penetration ability [30]. In the realm of armor protection, the formation and distribution of ASBs are closely related to the distribution of maximum shear stress in the target material during impact. Moreover, during ballistic impact testing, the existence of ASBs indicates that the material has experienced extremely high strains and temperatures in localized regions, which can lead to localized material failure and a reduction in armor effectiveness, as shown in Figure 2 [31]. Thus, it is essential to avoid the onset of adiabatic shear instability in armor protection applications [8,32,33]. Using a thick-walled cylinder (TWC) method to simulate the fragmentation process, multiple shear bands emerge and interact under high-strain-rate loading conditions, exhibiting self-organizing behavior. Simulation results indicate that the number and spacing of ASBs are closely linked to the strain rate and material properties. It is also noted that thermal softening plays a crucial role in the initial stages of shear instability, but damage or defects contribute significantly more to the evolution of shear bands [34]. The emergence of ASBs facilitates the occurrence of projectile fracturing.

The phenomenon of adiabatic shear instability predominantly manifests in metalworking processes [17], including cutting [35,36,37,38], forging [15,39], and severe plastic deformation (SPD) methods such as torsion [40,41,42,43]. During cutting operations, the formation of ASBs enhances cutting performance. In high-speed cutting, materials temporarily become brittle due to the establishment of these shear bands, facilitating easier cutting, which results in reduced cutting force and temperature. This instability addresses common issues associated with traditional cutting processes, such as built-up edges, tool wear, and workpiece surface damage [44,45,46,47]. The high strain rates and strain concentrations within the shear bands can lead to rapid crack propagation and material failure, as illustrated in Figure 2. In high-speed cutting, such phenomena contribute to chip formation, thus improving the cutting process [45]. Cylinder-shaped specimens were dynamically compressed to simulate the forging process, revealing shear band formation resulting from non-uniform deformation. Grain refinement and orientation changes occur within the shear bands, as shown in Figure 2, influencing the overall material properties, including strength and toughness. Additionally, the non-uniform internal deformation caused by shear band formation may lead to cracks or fractures during forging [39]. The mechanical properties of materials within shear bands often differ from those of the matrix material, and these discrepancies may affect the performance of the final product, necessitating their avoidance. Adiabatic shear instability is also critical in large plastic deformation processes. In studies on equal channel angular pressing (ECAP) of AZ31 magnesium alloy, the emergence of shear bands contributes to grain refinement. However, this can result in non-uniform deformation and localized stress concentrations, potentially diminishing the overall performance of materials, particularly concerning fatigue and fracture toughness. Furthermore, high strains and localized hardening within shear bands may complicate subsequent processing steps [41]. Consequently, the formation of shear bands during large plastic deformation presents both beneficial and detrimental aspects that must be weighed against specific requirements and processing conditions.

With the rapid development of advanced high-performance engineering applications in fields such as the aerospace and automotive industry, as shown in Figure 2, there is an increasing demand for high-strain-rate service performance of materials. In the aerospace sector, the dynamic properties of materials are directly related to the safety and reliability of aircraft. For instance, aerospace materials must possess high impact resistance to withstand various potential impacts encountered during flight, such as bird strikes and hail (as illustrated in Figure 2). High-strain-rate service performance ensures that materials can maintain their structural and functional integrity under high-speed impacts [48,49]. During automotive collisions, materials can experience very high strain rates, typically ranging from 10^2^ to 10^3^ s^−1^ [50], necessitating that automotive materials absorb energy and protect passenger safety during such incidents. Therefore, high-strain-rate service performance is critical for automotive materials, directly affecting vehicle safety in collision events. The phenomenon of adiabatic shear instability, which is commonly observed under high strain rates, is equally significant in automotive processes [50,51,52] (as demonstrated by the ASBs within automotive steel shown in Figure 2). A thorough understanding of the deformation and strengthening mechanisms of materials at high strain rates is essential for designing lightweight yet highly safe automotive components [53,54].

The phenomenon of plastic instability is not limited to any specific loading mode or alloy system; rather, it is widely distributed across a range of engineering issues. On the one hand, we must avoid the occurrence of this plastic instability, as shear localization is often accompanied by the accumulation of damage and a reduction in rheological stress, leading to further concentration of deformation. Continued stress can trigger the nucleation of micropores and microcracks, ultimately resulting in failure [6,39,59]. On the other hand, this phenomenon can also be harnessed to enhance the efficiency of machining processes and improve the penetration performance of projectile materials. Thus, elucidating the mechanism behind ASBs, evaluating material performance under high strain rates, and predicting the adiabatic shear instability phenomenon becomes particularly crucial.

## 2. Research Methods on Adiabatic Shear Instability

**Figure 3:**
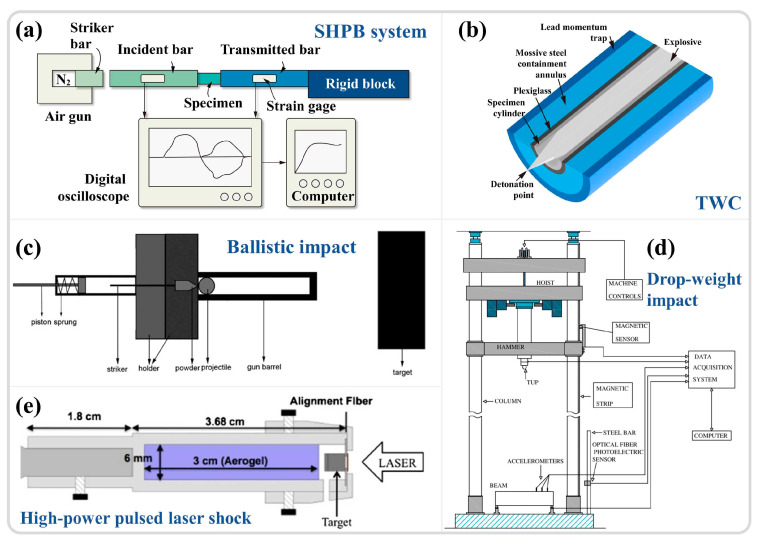
Schematic diagram of commonly used high-speed impact test techniques: (**a**) SHPB [26]; (**b**) TWC; (**c**) Ballistic impact [56]; (**d**) Drop-weight impact [65]; (**e**) High energy laser shock [71]. Reproduced with permission from Elsevier, Wiley, and open-access websites.

Adiabatic shear localization is closely associated with severe plastic deformation under high-strain-rate conditions. Since the phenomenon was discovered in 1878, various experimental techniques have been developed to investigate the dynamic mechanical behavior of materials. The use of integrated momentum capture devices, pulse forming equipment, strain measurement techniques [6], and more recently developed ultra-high-speed imaging devices and transient temperature measurement instruments [24,25] have made it possible to study these instabilities and even establish the spatial-temporal relationships in the adiabatic shear instability process. The most notable of these techniques include the split Hopkinson pressure bar (SHPB) [60,61,62], as evidenced by a literature review of research results from the past two decades, the TWC experiments [32,63], ballistic impacts [30,31], drop-weight tests [39,64,65], and laser shock compression experiments [66,67,68]. An overview of these commonly used high-speed impact experimental techniques is presented in Figure 3 [56,65,69,70,71].

### 2.1. High-Strain-Rate Experimental Method

**Figure 4:**
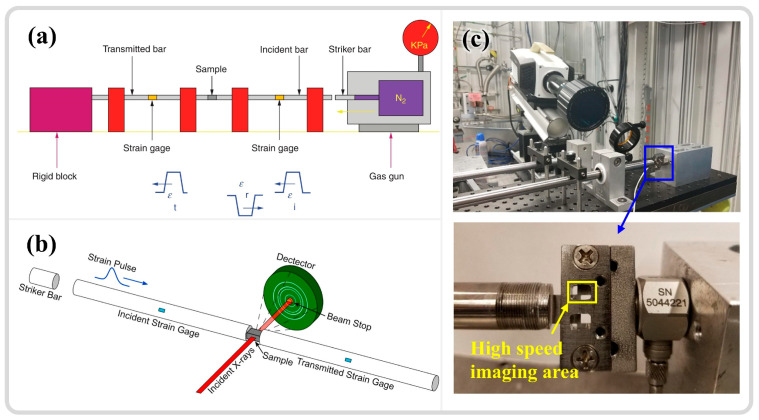
(**a**) Schematic illustration of the SHPB [74]. (**b**) Schematic diagram of the synchrotron X-ray radiation integration with SHPB [10]. (**c**) Integration of SHPB with infrared thermometry and high-speed imaging techniques, and further magnification of the high-speed imaging area. [76]. Reproduced with permission from Elsevier, Wiley, and Springer Nature.

The SHPB system primarily consists of a striker bar, an incident bar, and a transmission bar, as illustrated in Figure 4a [69]. The specimen is positioned between the incident rod and the transmission rod, with the load applied in the form of a stress wave. The strain rate is controlled by adjusting the air pressure in the striker bar’s gas chamber, with experimental strain rates ranging from 10^2^ to 10^4^ s^−1^ [72,73]. The relationship between the voltage recorded by the strain gauges on the incident and transmission rods and time (Figure 4a) is converted into relationships involving time, stress, strain, strain rate, and displacement through one-dimensional stress wave theory, using time as a bridge to infer the material dynamic mechanical behavior [11,74]. The scientific study of adiabatic shear localization ultimately aims to correlate changes in apparent strength, temperature increase in unstable regions, and the evolution of microstructures, even at the nanoscale substructure level [6]. As an ideal and reliable high-strain-rate loading technique, SHPB is extensively utilized to quantify the dynamic responses of various metallic materials under high strain rates. With advancements in instrumentation science and the integration of in situ time-resolved techniques (high-speed thermometry and imaging), this field continues to progress. Hudspeth et al. [75] combined the high-speed loading capabilities of SHPB with X-ray synchrotron radiation (Argonne National Laboratory, US), achieving a time resolution of 0.5 μs and a spatial resolution on the micron scale (Figure 4b). Magagnosc et al. [10] applied this technology to investigate the internal microstructural evolution of Ti-7Al under ASB conditions, observing that grain refinement may occur at approximately 1.1% strain after yielding. Nie et al. [76] integrated SHPB with infrared thermometry and high-speed imaging techniques, as shown in Figure 4c, achieving a spatial resolution of 1.6 μm and a time resolution of 200 ns. They studied the temperature distribution in the adiabatic shear zones of 7xxx and 6xxx aluminum alloys. These methods pave the way for more precise observations of ASB initiation and propagation. Given the high loading strain rate and relatively straightforward experimental setup of SHPB, as well as the reliable data collection, the analysis of original waveforms can indicate whether damage has occurred within the material [77], leading to its widespread application.

**Figure 5:**
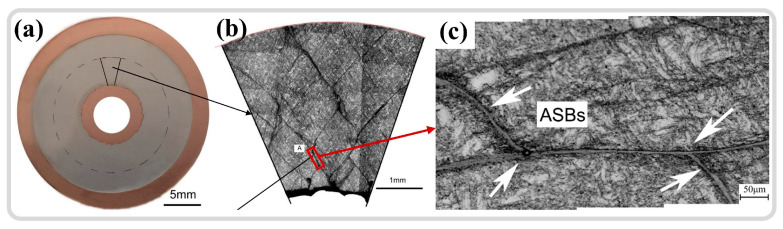
(**a**) An unetched cross-section of the cylindrical specimen from the TWC experiment. (**b**) Metallographic structure of the selected area in (**a**), which reveals a significant presence of ASBs internally. (**c**) A higher magnification view of the ASB in (**b**) [63]. Reproduced with permission from Elsevier.

The TWC explosion experiment serves as a method for studying the dynamic shear behavior of materials. The experimental setup primarily consists of a specimen cylinder surrounded by a thick steel ring, an acrylic tube to prevent collision between the specimen and the steel ring, and an external lead tube acting as a momentum trap, as shown in Figure 3b [70]. During the loading process, the specimen cylinder is filled with high-energy explosives (such as PETN), and the high pressure generated by the explosion causes the cylinder to expand and deform. By varying the explosive mixture and the thickness of the acrylic tube, the strain and strain rate of the specimen can be adjusted. Yang et al. [63] utilized the TWC experimental technique to develop multiple ASBs in ZK60 magnesium alloy (at a strain rate of 4.7 × 10^4^ s^−1^), as illustrated in Figure 5a–c, and conducted a systematic study on the competitive evolution, width, and distribution of the shear band propagation process. Nesterenko et al. [32] investigated the recrystallization behavior within the ASBs using TWC technology (at a strain rate of 3.5 × 10^4^ s^−1^), verifying through computational modeling that the changes in microstructures aligned with temperature predictions.

In summary, the advantages of this experimental method include the following: (1) The ability to halt experiments at different stages of shear band development, facilitating observations of their morphology at various stages. (2) The generation of numerous shear bands, allowing for quantitative statistical analysis. (3) The elimination of external stress concentrations, enabling the nucleation and propagation of shear bands to be governed by the microstructures of materials. However, it also presents certain disadvantages: (1) The initial complexity and inherent dangers associated with the use of high-energy explosives. (2) The roughness of the inner surface of the specimen cylinder (such as scratches) may serve as stress concentration sites, potentially affecting the experimental outcome.

**Figure 6:**
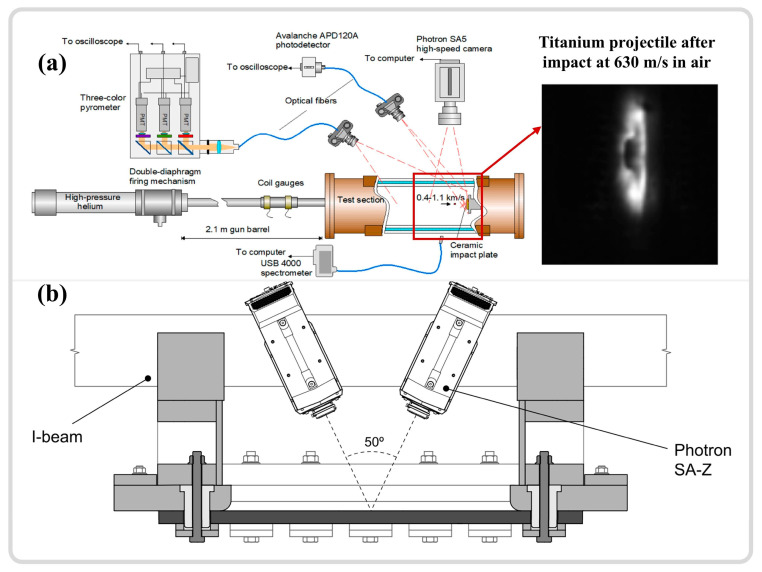
(**a**) Schematic representation of a 9 mm handgun bullet impacting a combat helmet analyzed using a high-speed camera [82]. (**b**) Schematic illustration of bullet impact combined with DIC [83]. Reproduced with permission from Elsevier.

As an important method for studying the dynamic response and failure behavior of materials under high-velocity impact conditions, this approach involves the impact of projectiles, such as spherical projectiles or those combined with handgun ammunition, rifle ammunition, and large-caliber projectiles [78,79], on target materials, as depicted in Figure 3c. This allows for the investigation of material deformation, crack propagation, and failure under high-strain-rate conditions. The strain rate range for ballistic impacts is typically very high, reaching up to 10^3^ to 10^6^ s^−1^. This method effectively simulates high-speed impact scenarios encountered in real applications [80], such as ballistic missiles and projectile impacts, making experimental results more applicable to actual situations. Moreover, various technologies have been developed in conjunction with ballistic impact experiments, including high-speed cameras (Figure 6a) [81,82], digital image correlation techniques (DIC) (Figure 6b) [83], flash X-ray cameras [84], and high-speed temperature measurement systems [78]. These advanced characterization methods enable researchers to analyze material dynamic strength, crack propagation characteristics, and energy absorption capacity with greater precision. Although ballistic impact experimental methods also have some drawbacks, such as complex operational procedures, high experimental costs, and challenges in data processing, they play an irreplaceable role in assessing the impact resistance and dynamic responses of materials.

Compared to the three experimental techniques mentioned above, the drop-weight test has a relatively lower strain rate, ranging from 10^−1^ to 10^3^ s^−1^, which is suitable for studying the dynamic behavior of materials under a relatively low-velocity impact [78]. This test involves dropping a heavy hammer from a certain height onto a clamped specimen, achieving dynamic deformation of the material, as shown in Figure 3d. The height of the drop determines the impact energy applied to the test material, which is then converted into important parameters, including force, velocity, displacement, energy, and time through the collection of electrical signals by sensors [65]. Sun et al. [39] utilized the drop-weight test (strain rates of 10^2^ to 10^3^ s^−1^) to study the shear instability behavior of pure titanium under dynamic compression, focusing on the evolution of microstructure and grain orientation during the formation of shear bands. Compared to other high-strain-rate testing methods, the drop-weight test is relatively simple and less expensive, yet it can provide valuable insights into the fracture toughness, deformation behavior, and failure modes of materials.

Laser shock compression technology focuses high-energy laser pulses onto the material surface, generating plasma and explosive pressure, thereby inducing shock waves within the material, achieving extreme compression, as illustrated in Figure 3e. This technique can attain extremely high strain rates (10^6^–10^10^ s^−1^) [85]. Zhao et al. [86] utilized a Janus laser to produce large-amplitude stress pulses of nanosecond duration (peak stresses of 45–50 GPa), investigating the amorphization of silicon carbide under high pressure. Meanwhile, Zhao et al. [87] conducted further research on the amorphization and nanocrystallization of germanium semiconductor materials during laser shock compression. Under a high shock stress of 33 GPa, a nanocrystalline structure formed on the germanium surface, accompanied by high-density nanotwinning. At a lower shock stress of 13 GPa, the germanium surface completely amorphized, with ASBs playing a significant role in the amorphization process, a viewpoint similar to studies on the high-strain-rate amorphization of boron carbide, where shear strain induces substantial lattice displacement leading to amorphization [66]. Laser shock technology provides extreme strain-rate conditions irreplaceable by other high-strain-rate experimental methods, while allowing precise control of experimental conditions, which is of vital importance to fields such as materials science and physics. The main drawbacks are the complexity and high cost of experimental equipment, and the challenge of data interpretation. Despite these challenges, laser shock compression technology remains a crucial method for exploring the behavior of materials under extreme conditions [88].

In summary, the SHPB is widely employed due to its simplicity, reliable data, and ease of integration with other advanced characterization techniques. With continuous development, the SHPB has evolved into the split Hopkinson tensile bar [89,90,91,92,93,94] and split Hopkinson torsion bar [95,96,97], facilitating tensile and torsional experiments at high strain rates. The TWC experimental technique generates a multitude of shear bands, facilitating quantitative statistical analysis of these bands. The impact of projectile testing closely mirrors practical applications, irreplaceably assessing the impact resistance of materials. Drop hammer tests primarily apply to the dynamic mechanical behavior of materials under low-velocity impacts. Laser shock compression offers extremely high strain rates crucial for condensed matter physics. Beyond these high-strain-rate experimental methods, numerous other excellent techniques exist as well, such as the Taylor impact [98,99,100,101] and the flying plate test [78,102].

### 2.2. Specimens for Shear Instability Research

Stress distribution, as a key factor in the plastic deformation process, is affected by the specimen geometry and dimensions. In high-strain-rate experimental techniques, the formation of shear bands additionally depends on the specimen geometry and material properties [6]. Based on the influence of specimen geometry, two types of shear localization (i.e., forced shear localization and spontaneous shear localization) are distinguished. Forced shear localization is commonly achieved with hat-shaped specimens, whereas symmetrical structures like cylindrical specimens typically exhibit spontaneous shear localization. Consequently, the specimens of various shapes in adiabatic shear instability research are specified below.

- Cylindrical specimen

**Figure 7:**
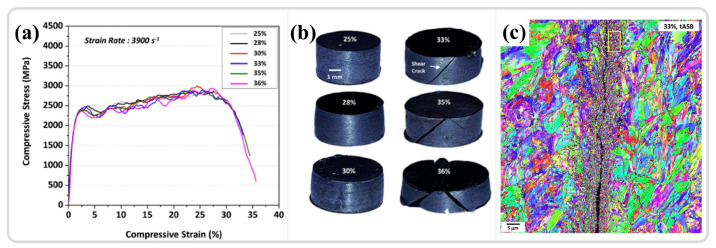
(**a**,**b**) The mechanical response and macroscopic morphology of cylindrical specimens under varying strains. (**c**) The orientation distribution results in the ASB region at a strain of 33% [103]. Reproduced with permission from Elsevier.

Cylindrical specimens, having the simplest geometric shape, are commonly utilized for studying the quasi-static and dynamic mechanical responses of various materials. The length of the cylindrical specimen, denoted as ‘l’, should be no greater than its diameter, ‘d’, typically set at a ratio of (0.5, 0.7, 1) to ‘d’. With these specimen dimensions, the time for stress waves to reciprocate within the specimen is relatively short, allowing for multiple reflections that tend to balance the internal stress and strain, thereby neglecting the effects of wave propagation. Jo et al. [103] employed cylindrical specimens in conjunction with the SHPB technique to investigate the microstructural evolution and formation mechanisms of ASB in high-strength armor steel. As illustrated in Figure 7a, strain control under constant strain-rate loading was achieved via a limiting ring. Cracks initiated in the cylindrical specimen at 33% strain, approximately oriented at 45° to the specimen axis, as shown in Figure 7b. The onset of plastic instability frequently correlates with the accumulation of damage, with cracks extending throughout the ASB and significant refinement of the microstructure within the shear bands from an initial 20 μm to 260 nm, as depicted in Figure 7c. Within the specimen, two shear bands commonly intersect at 45° to the axial direction [104,105,106]. Sun et al. [28] utilized cylindrical specimens to study the adiabatic shear sensitivity of tungsten-based alloys, discovering that the presence of high-density dislocations and twins within pre-deformed samples facilitated the onset of rotation dynamic recrystallization (RDR) mechanisms. The twins formed post-RDR enhanced stress concentration, making micro-damage more susceptible to nucleation and propagation.

Advantages of cylindrical specimens: (1) The onset of adiabatic shear instability is related to the material’s intrinsic properties and is unaffected by specimen geometry. (2) Dynamic mechanical curves can reflect the material critical strain and strain rate for adiabatic shear instability. (3) Specimens are easy to fabricate, saving costs. Disadvantages: (1) The adiabatic shear phenomenon in materials is localized, yet the specimen can continue to bear load post-occurrence. The stress–strain curves from experiments do not effectively represent the states at various stages of the adiabatic shear phase, making it challenging to depict the entire process from initiation to conclusion. (2) The ASBs in cylindrical specimens occur internally, complicating in situ time-resolved analysis.

- Hat-shaped specimen

**Figure 8:**
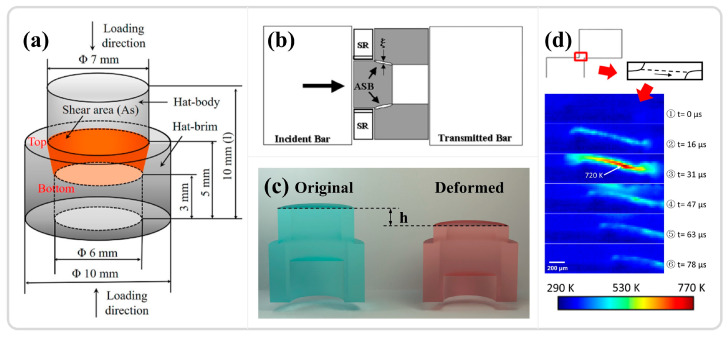
(**a**) Schematic illustration of the hat-shaped specimen, with orange indicating the region of forced shear [111]. (**b**) Control of strain through the use of a stopper ring [112]. (**c**) Diagram illustrating the control of strain by regulating the indentation depth of a protrusion [11]. (**d**) Measured temperature evolution in the shear region of a flat hat-shaped specimen [76]. Reproduced with permission from Elsevier and Springer Nature.

Hat-shaped specimens are commonly used in the study of adiabatic shear instability phenomena. These specimens transform the uniaxial stress state into a combined compression-shear or pure shear stress state through their geometry, confining plastic deformation within a pre-designed stress concentration zone. A minor compression increment achieves a significant shear strain, inducing the onset of ASB in the orange region depicted in Figure 8a. The compression increment of the protrusion of the hat-shaped specimen can be regulated by a stopper ring (as shown in Figure 8b) to control the deformation within the shear region, as illustrated in Figure 8c. Initially designed by Meyer and Hartmann et al. [107,108], the hat-shaped specimen has been extensively applied in the ASB studies of materials such as magnesium alloys [109], aluminum alloys [110], steel [111,112], and titanium alloys [113,114,115,116,117]. Typically, the protrusion of the hat-shaped specimen is slightly larger than the diameter of the specimen base, leading the forced shear region to bear the impact of compression loads in addition to shear stress. Minnaar et al. [118] reduced the protrusion to slightly smaller than the base diameter by modifying the specimen geometry to minimize the impact of compressive stress on testing, and they investigated the adiabatic shear behavior of various steels and titanium alloys. They concluded that the failure and ultimate fracture caused by plastic instability cannot be fully explained from a thermodynamic perspective, requiring additional considerations of microstructural damage mechanisms. Traditional cylindrical hat-shaped specimens also face challenges in in situ analysis, prompting the development of flat hat-shaped specimens. Combined with high-speed temperature measurement, these specimens facilitate the study of the relationship between temperature evolution and dynamic mechanical response in the shear region, as shown in Figure 8d, which is significant for elucidating and explaining the microstructural evolution within ASB [76,119,120,121].

Advantages of the hat-shaped specimen: (1) The specific specimen geometry enforces shear, allowing ASB to occur in the designated region, thus facilitating subsequent characterization analysis. (2) Due to its unique geometry, it can achieve extremely high local shear strain and strain rates, enabling shear localization phenomena even in ductile materials. (3) Loading under different stress states can be achieved by controlling the specimen dimensions. (4) The flat hat-shaped specimen facilitates in situ temperature measurement in the shear region. Disadvantages: (1) Compared to cylindrical specimens, it is more difficult to fabricate. (2) Although the obtained dynamic mechanical property curves can assess internal instability or damage in the sample, the stress–strain curves reflect a composite of the specimen geometry and material mechanical properties and cannot determine the material’s intrinsic performance.

- Shear compression specimen

**Figure 9:**
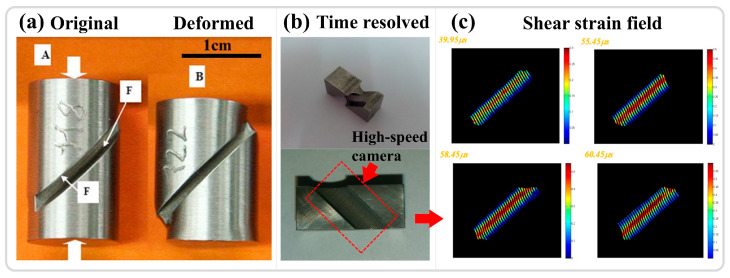
(**a**) Morphology of cylindrical SCS specimens before and after deformation [122]. (**b**) Cubic SCS specimens with marked high-speed camera and temperature measurement areas [25]. (**c**) Shear strain field distribution in the groove area of the SCS specimens at different loading times, as in (**b**) [25]. Reproduced with permission from Elsevier and Springer Nature.

The shear compression specimen (SCS), a commonly used specimen for studying adiabatic shear instability, typically consists of a cylinder or cube with two grooves inclined at 45° relative to the longitudinal axis, as shown in Figure 9a,b for cylindrical and cubic SCS specimens, respectively [25,122]. Under compressive loading, the designated area primarily undergoes shear deformation. Similar to the hat-shaped specimen, the onset of instability in the SCS specimen depends on the material’s intrinsic properties and the specimen’s geometric dimensions. The macroscopic morphology before and after deformation is illustrated in Figure 9a. The grooves in the shear region are exposed, facilitating time-resolved observation and analysis of the shear region, as demonstrated in Figure 9b with high-speed filming of the red-colored groove, and the results, as shown in Figure 9c, depict the variation of the shear strain field with time [25]. Zhang et al. [123] utilized SCS specimens in conjunction with high-speed infrared detectors to investigate the thermomechanical response and instability behavior of two different types of titanium alloys under high strain rates. The findings indicate that, prior to a significant drop in stress and the onset of adiabatic shear instability, the conversion ratio of plastic work to temperature differs from the traditionally assumed values of 0.9 or 1. The temperature rise in the adiabatic shear region is relatively limited and insufficient to trigger thermoplastic instability, which aligned with the research results of Guo et al. [24]. The advantages of the shear compression specimen include the following: (1) The groove design on both sides of the cylinder or cube enables high-speed imaging, thermometry, and other real-time characterization. (2) The geometric dimensions induce stress concentration in the designated area, facilitating the study of adiabatic shear instability phenomena. Its potential disadvantages are that, compared to traditional compression or tension specimens, the design and fabrication of SCS specimens may be more complex and time-consuming.

- Numerical simulation of adiabatic shear instability

In addition to the traditional high-strain-rate loading methods, the extreme conditions encountered during adiabatic shear instability deformation make it challenging for conventional experimental methods to capture and analyze the entire process in real time. Consequently, numerical simulation studies for adiabatic shear regions have emerged. An et al. [124] employed the crystal plastic finite element method (CPFEM) to simulate the dynamic compression, dynamic tensile, and shear deformation processes of nanocrystalline Cu-Al alloys. The results indicated that during high-strain-rate deformation, dynamic DRX leads to material softening through dislocation density reduction and grain refinement, thereby facilitating shear localization. Similarly, Nguyen et al. [125] utilized the CPFEM method to investigate the deformation behavior of single-crystal tantalum under dynamic loading. The extended dislocation density model accurately predicts the deformation behavior of single-crystal tantalum at varying impact velocities, aligning with experimental findings.

The CPFEM method is particularly suitable for studying the plastic deformation behavior of polycrystalline metallic materials. This method shows several advantages: firstly, based on a physical model centered around dislocation density, it can quite precisely depict the microstructural evolution of materials during high-strain-rate deformation. Secondly, its high accuracy enables the prediction of complex phenomena like shear localization, with results aligning well with experimental data. Additionally, it facilitates multi-scale analysis of high-strain-rate deformation in materials, bridging the macroscopic behavior of dynamic deformation with the evolution of microstructures. However, CPFEM also faces certain limitations. Its complex calculations demand substantial computing resources and time, particularly in high-resolution simulations. Furthermore, the model’s accuracy relies heavily on precise parameter calibration, necessitating extensive experimental data support.

Bronkhorst et al. [126] studied the adiabatic shear localization behavior of tantalum and 316L stainless steel by combining experimental and finite element methods. The results indicated that no shear bands were formed in tantalum, whereas the 316L stainless steel sample developed shear bands in the later stage. Simulation results revealed that the ASB temperature of tantalum reached 900K, while that of 316L stainless steel reached the melting temperature of 1670K in a short period of time. The simulation results fairly accurately predicted the experimental data, particularly the macroscopic behavior prior to material instability. Vishnu et al. [127] explored the effects of porosity, void size, and temperature softening on the formation and development of shear bands through three-dimensional finite element calculations and experimental verification. Voids facilitated dynamic shear localization, acting as preferential nucleation sites for shear bands, thereby accelerating the development of shear bands and altering their propagation direction.

The advantages of the finite element method include its ability to predict the deformation behavior of materials under complex loading conditions, particularly the response at high strain rates. Additionally, it allows for a detailed analysis of the distribution and evolution of stress, strain, temperature, and other parameters of the material. Compared to experiments, finite element numerical simulation offers significant time and cost savings, especially when exploring the effects of different parameters. Furthermore, the conditions of finite element simulations can be easily controlled, facilitating the repetition of experiments and verification. However, this method still has several shortcomings. Firstly, it relies on the constitutive model and material parameters. If the model is improperly selected or the parameters are inaccurate, it may lead to significant deviations in the simulation results. Secondly, simulation results are sensitive to grid density and geometric shape, and grid distortion may result in inaccurate outcomes. Under extreme conditions, such as high temperatures and high strain rates, the physical behavior of materials can be highly complex, and some models may not fully describe all phenomena [128].

Jiang et al. [129] conducted a study on the abnormal hardening and amorphous phenomena of face-centered cubic high entropy alloys under extreme uniaxial tensile conditions by combining molecular dynamics simulations with experiments. They found that in the extreme dynamic tensile process, as the strain increased, the alloy sequentially underwent mechanisms such as twinning, localization due to de-twinning, phase transformation, and coexistent amorphization. The reasons for the phase transformation and the presence of amorphous bands were elucidated through molecular dynamics simulations. Molecular dynamic simulation methods can effectively depict dynamic deformation behavior and microstructural evolution at the atomic scale. Additionally, this approach can also reveal physical processes such as stress, temperature, and configurational entropy in the dynamic deformation process. However, the sample size and simulation duration in molecular dynamics simulations are relatively limited, making it challenging to fully reflect the properties of bulk materials. Furthermore, the simulation results are also dependent on the interatomic potential function and model parameters used [130,131,132].

## 3. Various Deformation Mechanisms Within ASBs

### 3.1. Formation of ASBs

As previously mentioned, the phenomenon of adiabatic shear localization in materials under high strain rates is considered as a result of the interplay between strain hardening, strain-rate hardening, and thermal softening mechanisms [6,16]. During high-speed deformation, most of the plastic work is converted into heat stored within the material. Due to the rapid deformation speed in a very short time, there is virtually no heat exchange with the external environment, leading to an inevitable temperature rise during high-speed deformation, which results in the occurrence of thermal softening. Recht [133] defined a critical condition for adiabatic shear instability, with the strain hardening and strain-rate hardening terms being positive and the thermal softening term being negative. When the slope of the dynamic shear stress–strain curve equals zero, a catastrophic slip occurs. In addition to thermal softening, the softening effect of the microstructure also plays a significant role in the development of ASB. Giovanola et al. [134] reported that the decrease in load-bearing capacity in dynamic mechanical response is due to the process of void nucleation, and the growth of microvoids did not commence prior to the onset of shear instability. Therefore, the softening is attributed to the microstructural softening effect of microvoids rather than temperature elevation, aligning with the results reported by Bai and Xue et al. [135,136]. The nucleation of voids and cracks is the cause of the significant reduction in load-bearing capacity (stress collapse) of low-carbon steel and TC4 titanium alloy. The analysis of the adiabatic shear regions prior to failure using a transmission electron microscope (TEM)—employing a hat-shaped specimen with strain control by a stopper ring—indicates that DRX may precede the initiation and connection of microcracks and microvoids. This suggests that DRX acts as a microstructure inducing adiabatic shear instability, occurring prior to peak stress during the early stages of deformation, a phenomenon verified experimentally by Rittel and Magagnosc et al. [10,137]. The onset of DRX leads to local softening [6,138], promoting localized plastic instability within the shear regions and further encouraging the initiation of cracks and voids, resulting in a significant decrease in stress levels. Andrade et al. [139] modified the Johnson–Cook constitutive equation to include the stress collapse induced by DRX. With an appropriate parameter selection, the constitutive equation closely matches experimental results. This well-founded speculation also requires an increase in temperature to support the occurrence of DRX and subsequent ASB formation. While previous theories considered adiabatic shear instability as a sudden instability, it is more appropriately described as a phase process transitioning from nucleation to failure rather than a sudden change. Besides temperature support for the DRX process, the accumulation of deformation energy, or cold work stored energy, is also believed to dominate the formation of DRX and the subsequent localization of adiabatic shear [140]. The microstructural softening led by cold work stored energy along with the temperature rise during the high-speed deformation is the cause of ASB formation, a viewpoint similar to Guan et al.’s research on Ti1023 shear instability [11].

In summary, the effects of thermal softening on the formation of ASBs have been extensively studied through experiments and numerical simulations. Besides thermal softening, the microstructural softening induced by microvoids also significantly contributes to the initiation of ASBs. During shear deformation, the gradual nucleation, growth, and aggregation of microvoids and microcracks result in a sudden decrease in rheological stress [141]. The above discussion on softening aspects encapsulates the formation of ASBs. The strain hardening effect during adiabatic shear instability, as a competing factor to softening effects, is closely linked to the evolution of microstructures in shear bands. Besides the DRX phenomenon mentioned, twinning, phase transformation, and amorphization also significantly influence the initiation and propagation of ASBs within materials. Consequently, the deformation mechanisms within adiabatic shear zones will be summarized and discussed.

### 3.2. Grain Refinement Within ASBs

**Figure 10:**
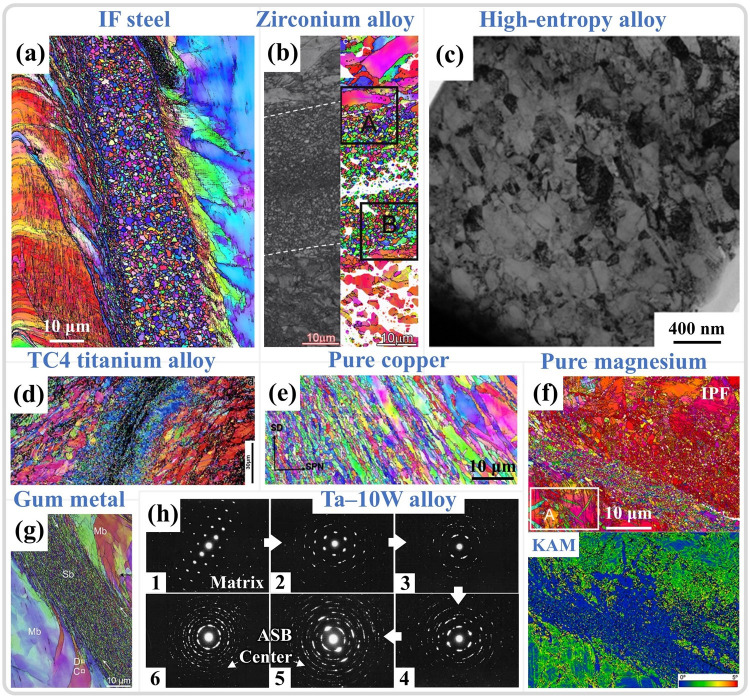
(**a**) IF steel ASB [112]. (**b**) Zirconium alloy ASB region Kikuchi band contrast (BC) and orientation distribution map [143]. (**c**) CrMnFeCoNi high-entropy alloy ASB center bright field (BF) morphology [62]. (**d**) TC4 titanium alloy ASB [146]. (**e**) Pure copper ASB [60]. (**f**) Pure magnesium ASB orientation distribution map and kernel average misorientation (KAM) distribution [109]. (**g**) Gum metal ASB [18]. (**h**) SAED results from the Ta-10W matrix to the ASB center, where 1–4 represent the SAED results outside, adjacent to, just inside, and close to the center of the shear region, respectively. Meanwhile, 5 and 6 depict the SAED results at the center of the shear region [148]. Reproduced with permission from Elsevier.

Extreme conditions within the ASB may induce various deformation mechanisms, which are dependent on the material type. As emphasized earlier, substantial grain refinement, a prevalent occurrence in the ASB, is pivotal for the initiation and progression of shear localization and the changes in apparent mechanical behavior. As depicted in Figure 10, the microstructures of adiabatic shear zones in steels [111,112,142], zirconium alloys [143,144], high-entropy alloys [62], titanium alloys [145,146,147], copper alloys [60], magnesium alloys [56,109], and tantalum–tungsten alloys [32,148] are summarized. The most distinctive characteristic is the equiaxed and fine-grained grains in the center of the ASB, with a relatively random orientation and limited strain, as seen in Figure 10f, where the ASB in a magnesium alloy exhibits a small KAM value. Additionally, the adiabatic shear zone can be divided into three parts based on its characteristics: (1) The equiaxed nanocrystalline core of the ASB; (2) The transition zone along the shear direction at the edge of the ASB; (3) The coarser matrix portion [11].

Substantial grain refinement within shear zones is widely observed in various metallic materials. Initially, through selected area electron diffraction (SAED) analysis of different regions within the ASB, it was determined that there are significant differences in the SAED patterns between the interior and exterior of the shear band [148,149]. Outside the shear band, the SAED results exhibit single-crystal diffraction spots. As the selected area moves toward the center of the ASB, the diffraction spots gradually transform into polycrystalline diffraction rings of nano grains (as shown in Figure 10h). In morphological observations, the grains at the center of the shear band appear as finely recrystallized equiaxed shapes. Meyers et al. [150] initially attributed the formation of nanoscale equiaxed grains to DRX. However, due to the extremely short deformation time under high strain rates, DRX relying on grain boundary migration mechanisms is difficult to achieve. Therefore, the mechanism of RDR relying on subgrain boundary transformation was adopted to explain the grain refinement phenomenon within the ASB [151]. This mechanism can be divided into the following five stages: (1) Initially, equiaxed grains have uniformly distributed dislocations. (2) As deformation continues, the originally equiaxed grains elongate and deform. (3) With continued deformation, dislocations gradually accumulate and tangle at subgrain boundaries, forming dislocation cell structures, and the elongated deformed grains are progressively subdivided. (4) The subgrain boundaries within the grains hinder the movement of dislocations, causing more dislocations to accumulate at the subgrain boundaries, gradually increasing the orientation difference between subgrains, and the elongated grains break up to accommodate strain. (5) The subgrains relax into equiaxed recrystallized grains by rotating 30° through subgrain boundaries, completing the formation of equiaxed nano grains through RDR [142]. It is typically combined with the RDR kinetics formula, as depicted in Equation (1), to verify whether DRX can be completed at the estimated temperature, with the meaning of the variables and parameters in the formula provided in Table 1 [11,26]. It should be noted that some variables and parameters in Table 1 do not have specific values because the variables, such as average subgrain diameter and absolute temperature, may vary greatly, depending on the experimental methods and materials. Specific experimental characterization results are needed, such as obtaining the average subgrain size through TEM, electron backscatter diffraction (EBSD), etc. The absolute temperature value is mostly determined through temperature estimation or even actual high-speed measurement, which will be analyzed and discussed in more detail in subsequent sections. In addition, parameters such as constants related to grain boundary diffusion and grain boundary thickness are closely related to the inherent properties of materials, and there are significant differences in numerical values among different materials. Therefore, a specific analysis is needed on the basis of different materials studied.

(1) $$\begin{array}{c} t=\frac{LkTf(\theta )}{4\delta \eta D_{\mathrm{bo}}\exp(-Q_{b}/RT)}\\ f(\theta )=\frac{3\tan(\theta )-2\cos(\theta )}{3-6\sin(\theta )}+\frac{2}{3}-\frac{4\sqrt{3}}{9}\ln(\frac{2+\sqrt{3}}{2-\sqrt{3}})+\frac{4\sqrt{3}}{9}\ln\frac{\tan(\theta /2)-2-\sqrt{3}}{\tan(\theta /2)-2+\sqrt{3}} \end{array}$$

**Figure 11:**
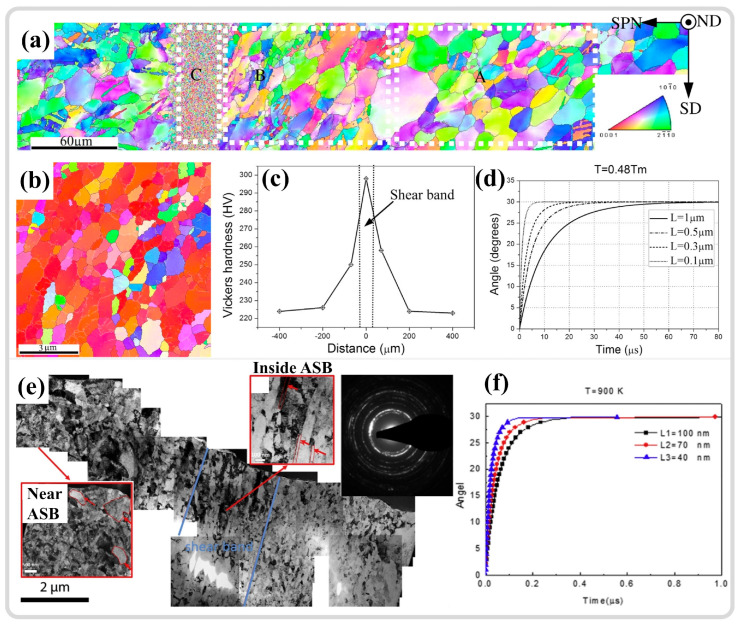
(**a**) Overall morphology of the ASB region in pure titanium. (**b**) Morphology of nano grains at the center of the ASB. (**c**) Distribution of Vickers hardness around the ASB. (**d**) Estimated time required for RDR at 0.48T_m_ [117]. (**e**) BF image morphology of the ultrafine-grained pure titanium ASB region and SAED results within the ASB. (**f**) Estimated time required for RDR at 0.46T_m_ [145]. Reproduced with permission from Elsevier.

Jiang et al. [117] employed SHPB combined with hat-shaped specimens to study the microstructural evolution of pure titanium ASB, indicating that the microstructure within the ASB consists of ultrafine grains with large-angle boundaries, with the average grain size decreasing from an initial 20 μm to 0.1–1 μm, as shown in Figure 11a,b. Additionally, the Hall-Petch effect and work hardening result in significantly higher hardness in the ASB region compared to the surrounding transition area and matrix, as shown in Figure 11c. With a ratio of 90% between plastic work and thermal conversion, the temperature in the ASB region is estimated to rise to 930 K (approximately 0.48 Tm (melting temperature)), corroborated by RDR kinetics calculations, which demonstrate that DRX can be completed within a loading time of 80 μs, as shown in Figure 11d, with equiaxed ultrafine grains being the product of DRX. Li et al. [145] investigated the microstructure of ultrafine-grained pure titanium ASB and observed that the initial 120 nm grain size structure can still undergo significant refinement in the ASB, with the grain size reaching approximately 40 nm, as shown in Figure 11e. The temperature in the ASB region rises to approximately 900 K (about 0.46 Tm), with the same conversion ratio of 0.9 used for temperature estimation, enabling RDR, as shown in Figure 11f. Such a temperature increase in the ASB is an important consideration for studying microstructural evolution mechanisms [114]. The temperature rise during high-strain-rate deformation could be described by the following equation [152],

(2) $$\Delta T=T-T_{0}=\frac{\beta }{\rho C_{v}}\int_{\gamma_{s}}^{\gamma_{e}}\tau \;d\gamma$$

where Δ*T* is the temperature increase caused by adiabatic heat, *T*_0_ is the initial temperature (293K), *β* is the ratio of plastic work converted to heat (Taylor-Quinney coefficient (TQC)), *ρ* is the density, and *C_v_* is the heat capacity.

**Figure 12:**
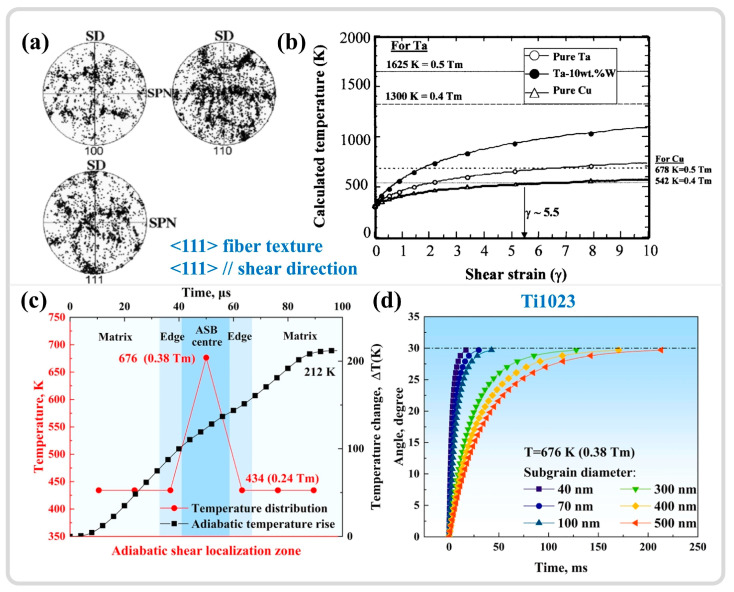
(**a**) Tantalum ASB center pole figure. (**b**) Temperature rise with increasing shear strain for pure Ta, Ta-10W, and pure copper calculated using the Johnson–Cook equation [148]. (**c**) Temperature distribution in the shear region of Ti1023. (**d**) Time required for RDR completion at 0.38Tm for different subgrain sizes in Ti1023 [7]. Reproduced with permission from Elsevier.

However, studies also indicate that DRX may not be completed during high-speed loading, and the substantial refinement of grains is a result of dynamic recovery (DRV). Pérez-Prado and colleagues [148] studied the microstructural evolution of the ASB in Ta-W alloys. While TEM analysis revealed grain refinement at the ASB center, a pronounced texture was observed, as shown in Figure 12a, which contrasted with the more random orientation of DRX grains. Furthermore, estimates of temperature increases in the ASB showed that under the same shear strain, Cu could reach 0.5Tm, but for pure tantalum and tantalum–tungsten alloys, this temperature increase was quite limited (with a 90% TQC), as shown in Figure 12b. The RDR mechanism cannot be executed during high-speed loading due to insufficient time and temperature to support subgrain refinement through rotational relaxation. DRV is a more protracted process than DRX, as it is completed only after the final step of RDR, releasing the strain energy within subgrains through rotation. However, in this study, grain refinement is more appropriately attributed to the segmentation and fragmentation of initial grains by subgrain boundaries. This conclusion also aligns with the views of Wang et al. [153] and Guan et al. [7,11], who suggest that the limited temperature increase in adiabatic shear zones (with a 90% TQC) precludes DRX; grain refinement is primarily caused by DRV driven by dislocation migration, as illustrated in Figure 12c,d. Therefore, a summary of the grain refinement phenomena and estimated temperatures within the ASB of various materials from the literature is presented in Table 2.

**Figure 13:**
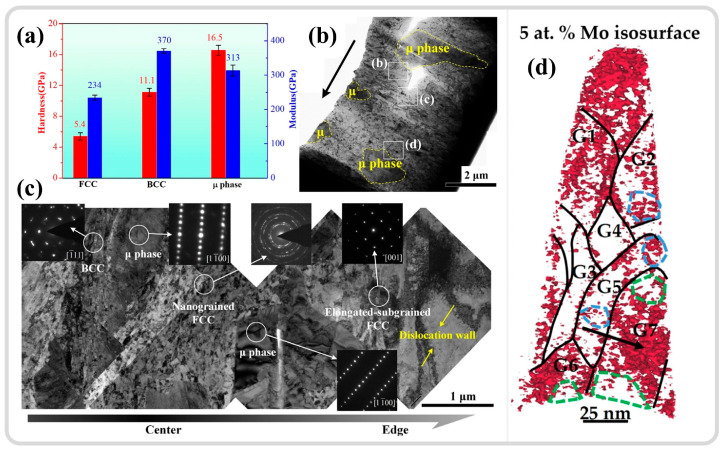
(**a**) Hardness difference between the matrix phase and precipitated phase within tungsten-based high-entropy alloys. (**b**) Macroscopic BF image morphology and the distribution of superhard μ phase within the ASB of tungsten-based high-entropy alloys. (**c**) BF image morphology and SAED results of different phases from the center to the edge of the ASB [30]. (**d**) APT Mo element 5 at.% isosurface distribution at the center of the ASB in dual-phase titanium alloys [159]. Reproduced with permission from Elsevier.

Liu et al. [30] investigated the adiabatic shear behavior of tungsten-based high-entropy alloys, revealing that the ultrahard precipitated phase, μ phase, induces strain gradients within the microstructure, as shown in Figure 13a,b. The high-density dislocations in the local regions between the precipitated phase and the matrix phase trigger the initiation of DRX, as depicted in Figure 13c, serving as a softening mechanism that facilitates the formation of shear bands. This parallels the findings of Zhu et al. [159], who utilized atom probe tomography (APT) to study the composition distribution within an ASB of a dual-phase titanium alloy and the DRX behavior during adiabatic shear instability. The results indicated that β-stabilizing elements (Mo/Cr) become sparse in certain local regions of β grains, leading to the formation of finer α grains, as illustrated in the green areas of Figure 13d. In contrast, in some regions of α grains, β-stabilizing elements are enriched, resulting in finer β grains, as shown in the blue areas of Figure 13d, thereby dividing the original grains into finer DRX grains. The difference in mechanical properties between the α and β phases may also contribute to grain refinement [160]. A slight difference from the former study is that the redistribution of elements leading to phase transformation results from the combined effects of local plastic deformation and adiabatic temperature rise. The DRX process is not only driven by dislocation accumulation but also significantly influenced by compositional redistribution.

In summary, the inhomogeneous microstructure induces strain gradients within microregions, which in turn trigger DRX, serving as a softening mechanism that promotes adiabatic shear localization [138]. Lieou et al. [4] investigated this through polycrystal plasticity thermodynamics, demonstrating that DRX provides a crucial softening mechanism to explain the stress drop during adiabatic shear instability. For homogeneous microstructures, grain refinement mechanisms in ASBs often coincide with temperature estimations, with most results around 0.5 Tm, suggesting that DRX is the grain refinement mechanism in ASBs. However, some studies indicate that the adiabatic temperature rise may be too limited to support DRX, suggesting that DRV is the grain refinement mechanism. It should be noted that the interpretation of temperature rises due to adiabatic effects relies heavily on the conversion of plastic work to heat. Moreover, the stage at which DRX occurs is problematic, as the softening effects of microstructures play a crucial role in the formation mechanisms of adiabatic shear bands. DRX might be a triggering factor for adiabatic shear instability, promoting the formation of ASBs. These findings suggest that DRX might precede the formation of ASBs. However, whether DRX can occur is closely related to the strain energy stored within the material and temperature [161]. Could this imply that the temperature rise to initiate DRX precedes the peak stress, i.e., ASB formation? Yet, these studies, which use adiabatic temperature rise estimations to explain grain refinement, might indirectly suggest that DRX occurs after ASB formation. This is because temperature estimations in adiabatic shear regions depend on the accumulation of plastic work, which is linearly related to temperature, with the maximum temperature occurring at the end of the deformation phase. This line of reasoning is also closely related to temperature and will be discussed separately in the subsequent sections on the temperature in adiabatic shear regions.

### 3.3. Phase Transformation Within ASBs

**Figure 14:**
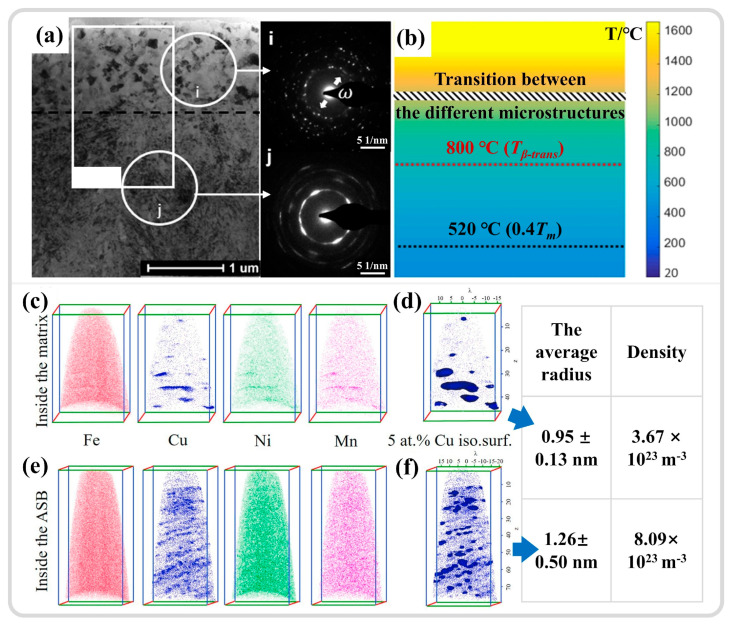
(**a**) BF image morphology of the adiabatic shear fracture zone and SAED. (**b**) Predicted maximum temperature during dynamic deformation in the area of (**a**), with β-transformation temperature and 0.4Tm temperature line indicated [21]. (**c**,**d**) Elemental distribution in the matrix and 5 at.% contour distribution of Cu element. (**e**,**f**) Elemental distribution in the ASB and 5 at.% contour distribution of Cu element, with statistics on precipitate size and density in the matrix and ASB [111]. Reproduced with permission from Elsevier.

In addition to the widespread grain refinement observed in the ASB, other deformation mechanisms are occasionally noted. Wang et al. [115] observed the presence of equiaxed α″ martensite in the ASB center of Ti55511, alongside general grain refinement. Calculations based on the Fourier heat conduction equation indicate that the temperature in the ASB cools from a peak of 1132 K to room temperature in just 18 μs, corresponding to a cooling rate of 4.7 × 10^7^ K/s, being significantly higher than that for α″ martensite formation in titanium alloys (1.4 × 10^4^ K/s). The formation of α″ martensite is attributed to the extremely high cooling rate. Guan et al. [7,26] discovered in their study of the adiabatic shear behavior in Ti1023 that the formation of α″ martensite is due to stress-induced martensitic transformation. α″ martensite acts as a transitional phase from β to α, and its appearance promotes grain refinement by segmenting β grains, aligning with Zafari and Xia’s perspective [20]. Choisez et al. [21] investigated the Ti-12Mo alloy with an initial microstructure comprising equiaxed β grains and ω_ath_ nano-precipitates, observing that the nano-precipitates dissolve into the matrix during quasi-static tensile testing but re-precipitated within the ASB during subsequent high-strain-rate deformation, as shown in Figure 14a. This indicates that the ASB region underwent significant temperature elevation and cooling, as depicted in Figure 14b, with the dissolution and re-precipitation of the ω_ath_ phase contributing to the stabilization of the microstructure within the shear band, preventing further expansion of the shear band and premature material fracture. Long et al. [111] studied the effect of Cu-rich nano-precipitates on the adiabatic shear behavior of high-strength steel, revealing that temperature elevation in the ASB region leads to the coarsening of nano-precipitates and the precipitation of new precipitates, which compensate for the strength reduction due to DRX. Nano-precipitates can absorb and dissipate impact energy, inhibiting the formation and development of ASB. Chen et al. [162] investigated the microstructural evolution of the Ti-6Mo-3.5Cr-1Zr alloy during the ASB process, revealing a pronounced strain hardening phenomenon during dynamic compression. This behavior is primarily attributed to the stress-induced ω phase transformation. Concurrently, the stress-induced ω phase content increased from 30.7% to 66.5% with increasing strain. The stress-induced ω phase bands hinder the formation and propagation of ASBs, thereby delaying material failure and endowing the alloy with a higher compressive fracture strain (approximately 34%).

In summary, numerous alloys demonstrate phase transformations within their ASBs, correlating with severe shear deformation and increased temperatures. The uniformly distributed nano-scale precipitated phases can absorb and dissipate impact energy during high-strain-rate service, effectively inhibiting the development or initiation of adiabatic shear instability. Additionally, the generation of phase transformation facilitates the effective partitioning of the matrix structure, thereby promoting grain refinement.

### 3.4. Twinning Within ASBs

**Figure 15:**
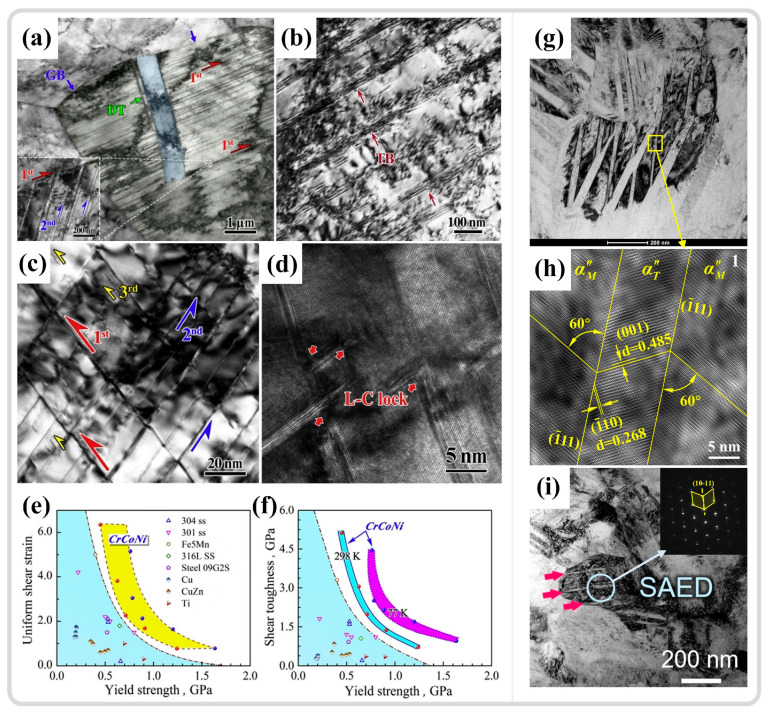
(**a**) Morphology of the uniform shear deformation zone under dynamic deformation at room temperature in BF imaging. (**b**) High-density dislocations at the twin boundaries and deformation twins at room temperature. (**c**) Multi-twinning grid morphology in the uniform shear deformation zone under dynamic deformation at low temperatures. (**d**) High-resolution transmission electron microscopy (HRTEM) image showing the Lomer–Cottrell lock in the shear deformation zone at low temperatures. (**e**,**f**) Comparison of the uniform shear strain and shear toughness of CrCoNi medium-entropy alloys with existing research results [163]. (**g**) High-density lath-like structure within the nano grains at the center of the ASB in Ti1023. (**h**) HRTEM image showing α″ martensitic nanotwinning in the rectangular region of (**g**) [7]. (**i**) {10–11}α nanotwinning morphology and SAED results at the center of the shear band in Ti1023 [11]. Reproduced with permission from Elsevier.

Twinning, as a rapid plastic deformation mechanism, can swiftly adapt to changes in external loads and coordinate internal deformation within materials. Thus, it is also present during the adiabatic shear instability process. Ma et al. [163] investigated the dynamic mechanical behavior and shear instability phenomena of CrCoNi entropy alloys at room temperature and low temperatures (77 K). During dynamic shear at room temperature, grain refinement, deformation twinning, and dislocation interactions are the primary strengthening mechanisms of the alloy, as shown in Figure 15a,b. Compared to room temperature, the twinning phenomenon is more pronounced at low temperatures, manifesting as higher twin density and multiple twin networks, as shown in Figure 15c. Dislocation slip is suppressed at low temperatures, making twinning a more effective plastic deformation mechanism, as shown in Figure 15d. Simultaneously, the interaction between twins and dislocations forms immobile Lomer–Cottrell locks and other dislocation locks [164,165], which, along with multiple twin networks, enhance the strain hardening capability of alloys. This strengthening mechanism aids in dispersing plastic deformation, thereby inhibiting the adiabatic shear localization and reducing the impact of thermal softening effects on the formation of ASBs. The combined effects of these deformation mechanisms provide the material with superior uniform shear deformation and shear toughness, as shown in Figure 15e,f. Li et al. [62] reported that the CrMnFeCoNi high-entropy alloy also exhibits excellent resistance to the formation of ASBs, forming nanoscale twins within DRX grains. The introduction of new interfaces at twin boundaries reduces the average free path of dislocation movement during deformation, enhancing the material work hardening and delaying the formation of shear bands. Guan et al. [7,11] observed in their study of the adiabatic shear behavior of Ti1023 that the emergence of nano-α″ martensitic twins (Figure 15g,h) and {10–11}α compressive twins (Figure 15i) in the ASB region may be responsible for inhibiting further stress reduction after adiabatic shear instability. The dynamic Hall-Petch effect increases the material work hardening capability, leading to a stable phase in the stress–strain curve after stress collapse. In summary, twin shear aids materials in adapting to high-strain-rate deformation processes, while the boundaries introduced by twinning further enhance the material uniform deformation capability. Twin formation, as an energy release mechanism, can effectively help materials adapt to external loads in a short time so as to avoid crack formation and propagation [166]. The emergence of twinning effectively suppresses adiabatic shear instability phenomena, improves the material resistance to the formation of ASBs, and enables materials to exhibit superior mechanical properties at high strain rates.

### 3.5. Amorphization Within ASBs

**Figure 16:**
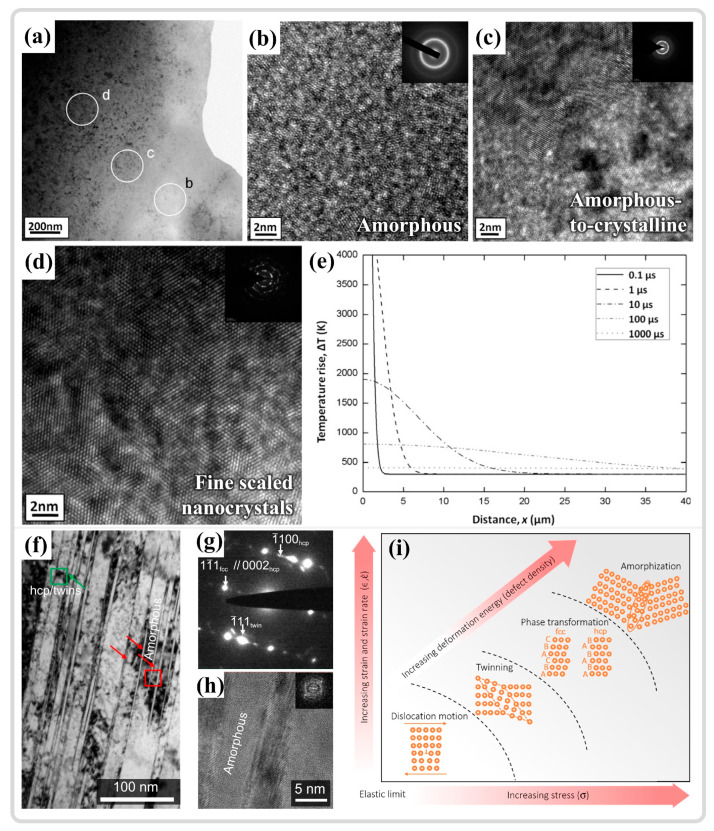
(**a**) BF imaging morphology of the ASB. (**b**–**d**) HRTEM images and the corresponding SAED results of the amorphous region, the transition region from amorphous to crystalline, and the fine scaled nanocrystals region in (**a**), respectively. (**e**) Temperature distribution near the adiabatic shear zone as a function of distance in the shear band [167]. (**f**) BF images of the twins, hcp phase, and amorphous phase near the ASB in the CoMnFeCoNi high-entropy alloy. (**g**) SAED results indicating the presence of the hcp phase and twins. (**h**) HRTEM and Fourier transform results of the amorphous region. (**i**) Graded deformation mechanisms in CrCoNi-based high-entropy alloys—elastic deformation, dislocation migration, twinning, phase transformation, and eventual amorphization—where triggering the next mechanism requires the generation of additional defects [9]. Reproduced with permission from Elsevier and open-access websites.

Meyers et al. [142] reported the existence of amorphous regions within the ASB of AISI 304 stainless steel and posited that the substantial temperature increase and rapid cooling rates in the ASB contribute to the formation of this non-equilibrium structure. Li et al. [167] observed the co-existence of amorphous and nanocrystalline structures in the ASB center of TWIP steel, as depicted in Figure 16a. Figure 16b–d presents the HRTEM and the corresponding SAED results in the amorphous region, the transition zone between amorphous and crystalline phases, and the nanocrystalline region, respectively. The HRTEM results of the amorphous region exhibit a typical maze-like pattern, while the SAED reveals a more pronounced dispersed amorphous ring, distinct from the polycrystalline diffraction rings shown in Figure 16d. These findings confirm the co-existence of amorphous and nanocrystalline structures. Additionally, the temperature evolution near the shear band, illustrated in Figure 16e, suggests that melting may have occurred within the ASB and subsequently cooled at an extremely rapid rate, potentially explaining the presence of amorphization within the ASB. Zhao et al. [9] also noted this phenomenon in the ASB of CoMnFeCoNi high-entropy alloys, where amorphization took place in regions rich in defects such as stacking faults, twins, and interfaces of hcp phase transitions, as shown in Figure 16f–h. Theoretically calculated deformation within the ASBs leads to an elevation in defect density, thereby increasing the material energy content. Upon reaching a critical defect density, the FCC structure transitions to the HCP structure, ultimately leading to amorphization, as shown in Figure 16i. Contrary to earlier research, amorphization is a solid-state process that takes place well below the material melting point and should be more accurately defined consequently induced by high-density defects [66,87]. In the context of adiabatic shear instability, the amorphization process releases high-density defects such as dislocations, stacking faults, and twins, which inhibits the initiation of microcracks by reducing the stress generated. Moreover, the enhanced hardness of the amorphous region enables the material to absorb more strain energy under extreme deformation conditions, thereby improving the material impact resistance.

## 4. Temperature Evolution in Adiabatic Shear Region

Through a summary and discussion of various deformation mechanisms that may occur within ASBs, it is noticed that temperature consistently acts as a primary factor influencing the microstructural evolution within these bands. Estimates of temperature within ASBs validate the mechanisms of grain refinement and phase transformation. A series of research results involves the conversion of plastic work into heat, typically using a conversion ratio of approximately 0.9, as shown in Table 2. How close is this temperature estimation to the actual temperature increase? Is there a mechanism that may affect the rise in temperature during the adiabatic shear instability process, and does this temperature rise correlate positively with the accumulation of plastic work as estimated? Furthermore, according to the “thermal plastic instability theory”, temperature increases should occur prior to the formation of ASBs, where the competition between thermal softening, strain hardening, and strain-rate hardening ultimately leads to catastrophic stress collapse, marking the onset of shear localization. At what stage of mechanical response does the temperature rise actually occur? Before addressing these questions, it is essential to elucidate the sources of temperature.

**Figure 17:**
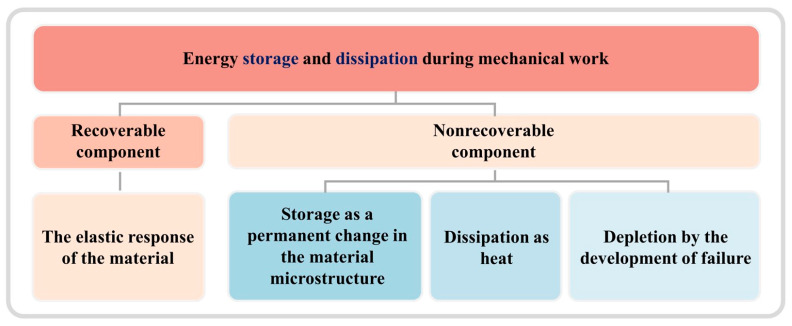
Schematic diagram of the relationship between plastic deformation and energy storage and dissipation.

During the process of plastic deformation, the work caused by external forces can be divided into two parts, as shown in Figure 17: (1) The recoverable part, which corresponds to the material elastic mechanical response; (2) The non-recoverable part, which primarily involves the evolution of the material microstructure during plastic deformation, with the defect-related energy such as dislocation slip, twinning, phase transformation, and amorphization being stored within the material. In fact, most of the energy associated with plastic deformation is typically dissipated in the form of heat. Additionally, there are consumption aspects related to failure development, such as the initiation and propagation of cracks and the coalescence of voids [168]. From the discussion of mechanical energy storage and dissipation during plastic deformation, it is evident that a conversion ratio of 0.9 may not be sufficiently rigorous. As illustrated in Figure 17, the temperature increase caused by energy dissipation is closely related to the storage of defects within the material and the development of failure. Therefore, the conversion of plastic work to heat should not be arbitrarily assigned a conversion ratio of 0.9 for all materials. In other words, different materials, crystal structures, loading methods, and deformation conditions can lead to variations in the choice of internal deformation mechanisms. This variation further affects the proportion of energy dissipated as heat, subsequently influencing the actual temperature increase.

**Figure 18:**
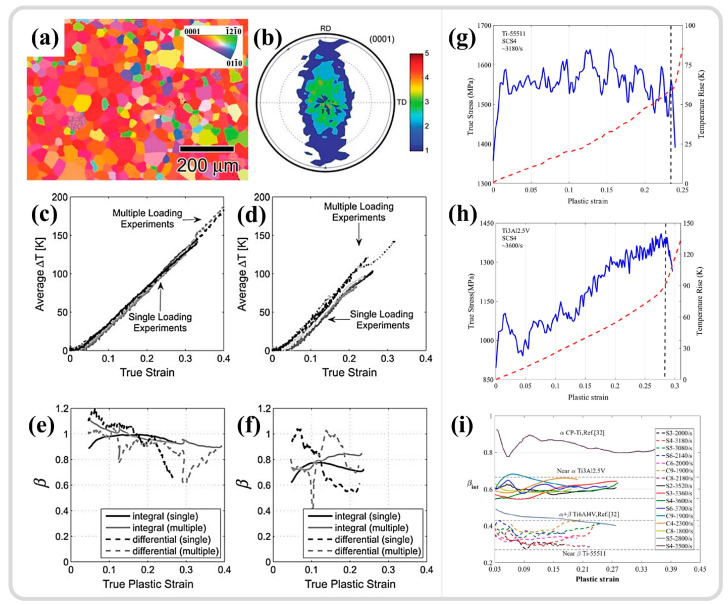
(**a**,**b**) The initial microstructure of Zr and the XRD macrotexture, respectively. (**c**,**e**) The temperature changes and the thermal–mechanical conversion ratio during compression along the RD direction. (**d**,**f**) The temperature changes and the thermal–mechanical conversion ratio during compression along the ND direction [169]. (**g**,**h**) The dynamic compressive mechanical response results (blue) and high-speed temperature measurement results (red) for the Ti55511 and Ti-3Al-2.5V alloys, respectively. (**i**) The thermal–mechanical conversion ratios for various materials at different strain rates [123]. Reproduced with permission from Elsevier and Springer Nature.

Padilla et al. [169] investigated the mechanisms of plastic deformation and energy dissipation effects of Zr (HCP) under high-strain-rate conditions. The change of temperature during high-speed compression was measured using the combination of SHPB and high-speed temperature detectors, focusing on the thermal dissipation during the plastic deformation process. The initial microstructure exhibited a distinct texture with the basal plane parallel to the normal direction (ND) of the rolled sheet, as shown in Figure 18a,b. Different deformation mechanisms were activated by compressing in various directions along the rolling direction (RD) and ND, with actual temperature measurements taken. The results demonstrated that samples compressed along the RD showed nearly complete conversion of plastic work into heat, with a conversion ratio approaching 1, as illustrated in Figure 18c,e. A significant number of deformation twins were present in these samples. In contrast, samples compressed along the ND exhibited a relatively lower conversion ratio, seen in Figure 18d,f, suggesting potential for energy storage; however, further analysis revealed that it was due to inhomogeneous deformation and localization of strain. Overall, the primary mode of energy dissipation for Zr under high-strain-rate conditions remains thermal. While the presence of deformation twinning alters the microstructure, its impact on energy storage appears limited, which is possibly more associated with strain inhomogeneity. Mason et al. [170] utilized a similar SHPB and high-speed temperature measurement approach to study the ratio of plastic work converted to heat in various materials, including 2024 aluminum alloy, 4340 steel, and Ti-6Al-4V titanium alloy. Their findings indicated that the β value (conversion ratio) for both 2024 aluminum alloy and 4340 steel increases with strain, ultimately reaching the commonly accepted range of 0.85–1.00. The Ti-6Al-4V titanium alloy exhibited different trends across various strain stages. At high strain rates, the β value showed significant dependence on the material strain. For the strain-rate insensitive materials, such as 2024 aluminum alloy and 4340 steel, the β value varied little across different strain rates. Conversely, strain-rate sensitive materials like Ti-6Al-4V showed a more complex relationship with strain and strain rate. Zhang et al. [123] investigated the thermomechanical conversion ratios of near α titanium alloy (Ti-3Al-2.5V) and near β titanium alloy (Ti55511). The results indicated that the temperature rise for both alloys prior to macro-load drop was relatively moderate, insufficient to induce thermoplastic instability, as shown in Figure 18g,h. Extensive twinning was observed in Ti-3Al-2.5V, while no twinning was noted in Ti-55511. The β value for Ti-3Al-2.5V was approximately 0.6, while that for Ti-55511 was around 0.35, as depicted in Figure 18i. This further suggests that the twinning deformation mechanism has a limited role in energy storage [171]. These findings align with those of Rittel et al. [172], where the thermomechanical conversion ratio β varies significantly with different materials rather than assuming a fixed value of 0.9. Additionally, different deformation modes—tension, compression, and shear—affect the thermomechanical conversion ratio by influencing the deformation mechanisms. The actual temperature measurements indicated a mildly continuous rise in overall temperature until the end.

**Figure 19:**
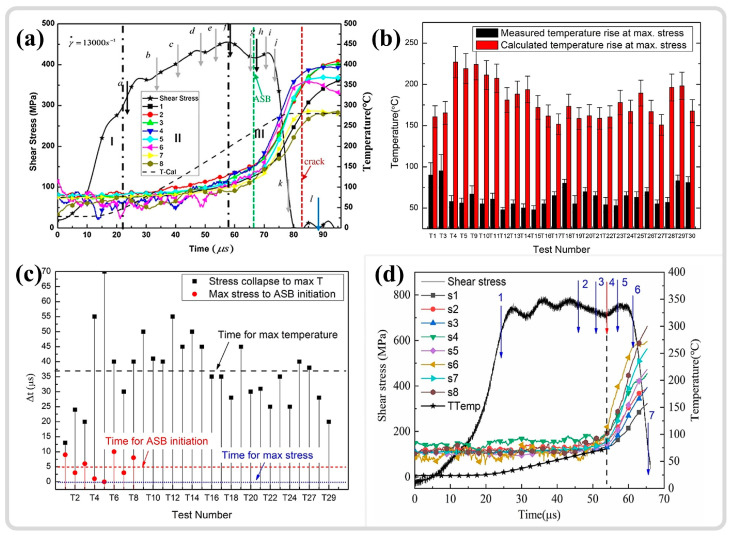
(**a**) The measured temperature and mechanical response results in the adiabatic shear zone. (**b**) A comparison between the measured temperature and estimated temperature at peak stress. (**c**) The chronological sequence of typical events during dynamic shear instability, where the results indicate that the peak shear stress occurs first, followed by the initiation of ASB, with the maximum temperature rise occurring last [24]. (**d**) The measured temperature and mechanical response results in the adiabatic shear zone [25]. Reproduced with permission from Elsevier and American Physical Society.

The thermal–mechanical conversion ratio is not the conventional fixed value of 0.9, as previously estimated, but rather varies due to the inherent characteristics of the material, the deformation mechanisms under different deformation modes, strain rates, and even the amount of strain; it is a constantly changing value [173]. Additionally, actual temperature measurements have yielded results that contradict traditional views, indicating that the temperature increase associated with adiabatic shear instability is relatively limited. This raises questions about whether this viewpoint undermines the explanation of how thermal plastic instability contributes to the formation of ASBs. If the temperature increase before the occurrence of adiabatic shear instability is moderate, the effects of thermal softening may be negligible. Guo et al. [24] conducted high-speed temperature measurements on numerous samples and illustrated their findings in Figure 19a, showing that the temperature increase prior to the localization of adiabatic shear is gradual, with significant temperature elevation occurring only after ASB formation, and this increase is limited (peaking at approximately 0.2Tm). There is a considerable discrepancy between the actual measured temperature at peak stress and the estimated temperature, as shown in Figure 19b. Statistics from multiple experimental groups indicate that during the process of adiabatic shear instability, the peak shear stress appears first, followed by ASB formation, with peak temperature being the last to manifest. Zhu et al. [25] obtained similar results in their high-speed temperature measurements on the Ti-6Al-4V alloy, as depicted in Figure 19d.

## 5. Summary and Prospect

### 5.1. Summary

The increasingly stringent demands of industrial applications in extreme environments impose higher requirements on materials for safe service under high-strain-rate conditions. The phenomenon of adiabatic shear instability, which is highly prone to occurrence under high-strain-rate conditions, often leads to catastrophic failure. Therefore, studying the mechanisms of this plastic instability is of vital importance for enhancing the performance of materials. This review organizes and analyzes literature related to ASB over the past two decades, comprising over 3400 papers. It covers various aspects of this plastic instability phenomenon, including the discovery and early theories of adiabatic shear instability, the fields in which the adiabatic shear phenomenon is widely observed, methods for conducting high-strain-rate experiments, the different deformation mechanisms within the ASB, and the temperature evolution in the shear zone. The following are the main aspects evaluated:

1. The application of ASB: The phenomenon of adiabatic shear instability is prevalent in metalworking and high-strain-rate service fields. In high-velocity impact or during the penetration of projectiles, it is crucial to suppress this instability to prevent deformation or even fracturing by the projectile, which can adversely affect its penetration capability. On the other hand, this instability can also be leveraged to maintain a sharp shape of the projectile, enhancing its penetration ability. In armor protection, it is necessary to avoid the occurrence of such phenomena to improve defensive capabilities. In metalworking, high-speed cutting processes can take advantage of this effect to enhance cutting efficiency and reduce cutting forces and temperatures, thereby improving operational efficiency. During forging, it is essential to prevent the emergence of this phenomenon, as it indicates impending failure. In the context of SPD, the influence of ASB must be carefully weighed. While the formation of shear bands can assist in grain refinement, it may also lead to microstructural inhomogeneity. In high-strain-rate service environments, such as aerospace and automotive industries, avoiding this instability is vital, as it directly impacts the safety and reliability of aircraft and motor vehicles.
2. High-strain-rate experiments: The SHPB method is widely used due to its simplicity, reliable data, and compatibility with various advanced characterization techniques such as high-speed imaging, temperature measurement, synchrotron radiation, and DIC. The TWC experimental approach can generate a large number of ASBs, which is crucial for quantitative statistical analysis of ASBs. Ballistic impact testing closely resembles real-world applications and plays an irreplaceable role in evaluating the impact resistance and dynamic response of materials. Drop-weight tests are more suitable for studying the dynamic response of materials under low-speed impacts. Laser shock compression provides extreme strain rate conditions that are unmatched by other high-strain rate methods, serving as an essential tool for exploring material behavior under extreme conditions. Beyond high-strain-rate methodologies, the geometry of the samples is equally critical. Cylindrical samples that undergo spontaneous shear localization effectively reflect the true performance of the materials. Hat-shaped samples that undergo forced shear localization and SCS samples induce ASBs in designated areas through their unique geometries, allowing for more targeted research on the phenomenon of adiabatic shear instability, thus finding widespread applications. Due to the extreme conditions of high-strain-rate deformation processes, numerical simulation methods such as finite element, crystal plasticity finite element, and molecular dynamics have been widely applied. At the same time, due to their high accuracy, they possess the advantage of predicting adiabatic shear localization and even simulating the microstructural evolution within ASB at the atomic scale. Therefore, they play an important role in predicting the service performance of materials at high strain rates and deepening the understanding of adiabatic shear instability phenomena.
3. The formation of ASB can be roughly categorized into two main theories. The first is the thermal softening or thermoplastic instability theory, which posits that under conditions of high-strain-rate loading, there is no thermal interaction between the localized deformation zone and the external environment. This situation causes most of the plastic work to convert into heat, stored internally within the material, leading to a significant rise in temperature. As the thermal softening effect gradually becomes predominant, ASB formation occurs when the slope of the stress–strain curve reaches zero. The second theory is the microstructural softening theory, which asserts that the softening effects at the microstructural level also play a crucial role in adiabatic shear instability. The nucleation, growth, and aggregation of microvoids and cracks contribute to a stress collapse phenomenon. The DRX phenomenon is often regarded as a softening factor that induces the occurrence of adiabatic shear instability and consequently leads to the formation of ASB. Furthermore, the evolution of various microstructures within the adiabatic shear zone cannot be overlooked in its influence on either promoting or suppressing the formation of ASB.
4. Microstructure evolution within ASB:
  (1) The phenomenon of grain refinement, recognized as the most significant feature in ASB, has been extensively studied. Most studies on estimating the temperature in ASB regions and calculating RDR kinetics support the mechanism of DRX responsible for grain refinement. However, some studies indicate that the temperature increase within ASB is limited, suggesting that grain refinement may be predominantly driven by dislocation migration or DRV. A stress gradient caused by an inhomogeneous microstructure may locally induce DRX in the material.
  (2) Phase transformation within ASB, characterized by fine and dispersed phases, aids in the absorption and dissipation of impact energy, thereby suppressing the formation of ASB. Simultaneously, the partitioning of the initial structure during phase transformation promotes further refinement of microstructures.
  (3) Twinning, as a rapid plastic deformation mode, adapts better to high-strain-rate loading environments compared to dislocation slips. The generation of twin boundaries and their interaction with dislocations enhance the material’s ability for uniform deformation, and the presence of twinning can effectively inhibit the occurrence of ASB.
  (4) The emergence of amorphization typically indicates a significant rise in temperature within ASB and, subsequently, rapid cooling. However, some studies suggest that amorphization may result from high-density defects induced under extreme deformation conditions within ASB. Additionally, the process of amorphization aids in defect release, contributing to enhanced impact resistance of the material.
5. Temperature within the ASB: The increase in temperature is related to the ratio of plastic work to heat dissipation during the deformation process, which is influenced by the microstructure in the material and the development of failure. The thermal–mechanical conversion ratio varies significantly in different materials rather than the presumed constant value of 0.9. Different deformation mechanisms, such as twinning, typically store lower energy levels, and both strain rate and total strain can impact the conversion ratio. The measured results for temperature in the ASB region indicate that the temperature rise in the adiabatic shear zone is limited, while significant temperature increases occur after the formation of ASB. These findings challenge traditional explanations of thermoplastic instability in relation to the formation of the ASB.

### 5.2. Prospect

A review of the field of adiabatic shear instability reveals that new research findings continue to emerge, uncovering some groundbreaking discoveries and offering insights into unresolved challenges. The steadily increasing number of publications each year also indicates the vitality of this field. We summarize the outstanding issues that still need to be addressed into two main categories:

First, modeling and estimating the temperature in the adiabatic shear zone, as well as actual measurements, are closely related to the storage and dissipation of mechanical energy during deformation. Dislocation slip, as the primary deformation mechanism under high strain rates, serves as a significant source of heat. However, other deformation mechanisms, such as twinning, have a more limited energy storage capacity. Therefore, the presence of different deformation mechanisms results in a varying ratio of mechanical energy storage to dissipation rather than the traditionally considered fixed value of 0.9. Additionally, different deformation modes (tension, compression, shear) also significantly impact the conversion rate of plastic work. Accurate measurement and estimation of temperature changes under high-strain-rate deformation conditions require further research, necessitating a more precise characterization and analysis of the physical mechanisms behind energy dissipation.

Due to the high-strain-rate conditions, the entire deformation process occurs on a microsecond scale, yet the formation of ASB remains a process from instability to localization. Most studies have focused on characterizing and analyzing samples post-loading and deducing the possible mechanisms involved in this process. Therefore, it is essential to identify various deformation mechanisms that influence the formation, propagation, and final fracture at different stages of shear localization, integrating apparent mechanical responses with microstructural changes. The effects of multiple deformation mechanisms on the formation and development of ASBs are still unclear and require further investigation. Additionally, these two aspects are not independent; temperature changes during high-strain-rate deformation can alter the stacking fault energy of the alloy system, consequently impacting the evolution of the deformation mechanisms. This necessitates a deeper integration of experimental observations and theoretical models to advance the understanding of the phenomenon of adiabatic shear localization.

The increasingly advanced analytical characterization methods and more realistic simulation techniques have deepened our understanding of the instability phenomena occurring in extreme deformation processes such as high-velocity impact, explosion, and penetration. Among the analytical characterization methods, EBSD, as a powerful probe integrated into scanning electron microscopes, plays an increasingly important role in the analysis and characterization due to its advantages, including flexible sampling areas, simple characterization experiments, high data accuracy (related to the selection of EBSD step size), and the ability to collect a large amount of crystallographic information in a short time. However, it is worth noting that EBSD still faces challenges in analyzing large deformation areas, making the analysis of ASBs with some light elements difficult. To address this, Transmission Kikuchi diffraction (TKD), also known as tEBSD, has emerged. Compared to the traditional EBSD, TKD significantly enhances spatial resolution, reducing the step size from approximately 0.04 μm in the conventional EBSD to 2 nm (axis TKD technology). Additionally, due to the improved spatial resolution, TKD can be used to better analyze the microstructure of large deformation areas, providing excellent characterization capabilities for the fine structures of ASB central regions. Transmission Electron Microscopy (TEM) has been widely applied. With the development of new characterization techniques such as HRTEM, scanning transmission electron microscope (STEM), and high-angle annular dark field (HAADF), it plays an increasingly important role in this research area, enabling atomic-scale analysis of the ASB region. The focused ion beam (FIB) method, which allows for sample extraction from extremely small areas, enables more precise sampling and subsequent analysis of adiabatic shear regions that are typically only on the micron scale, and it can easily integrate with TEM and TKD techniques for more accurate characterization. In the realm of simulation analysis, contributions from crystal plasticity and molecular dynamics are also on the rise. In the discussion regarding the temperature of the adiabatic shear region, the possibility of ultra-high-speed imaging and actual temperature measurement in these regions has become feasible with the continuous advancement of instrument science. The development of this series of analytical techniques will further deepen our understanding of the instability phenomena associated with adiabatic shear, clarifying the entire process from the onset of adiabatic shear to fracture, thereby providing a more robust theoretical foundation for enhancing the performance of materials under high-strain conditions.

Finally, a necessary condition for adiabatic shear instability to occur is that the stress–strain curve reaches a peak and then decreases with subsequent strain increase. In the previous discussion on the formation mechanisms of adiabatic shear bands, the instability phenomenon was primarily attributed to thermal softening due to temperature rise and microstructural softening effects resulting from factors like DRX and microcrack initiation. Since the onset of instability is due to the softening effect overcoming the mechanical strengthening mechanism, the question remains if we can delay or even avoid the occurrence of adiabatic shear by ensuring that the alloy exhibits a greater strain hardening ability in service. High entropy alloys exhibit higher work hardening ability under dynamic deformation conditions compared to traditional metals and alloys, owing to their pronounced lattice distortion, compositional fluctuation, and short-range ordering. This can be attributed to the diverse deformation mechanisms of high entropy alloys, indicating that they possess superior resistance to adiabatic shear instability compared to traditional alloys. However, plastic deformation can introduce defects within the material, which accumulate to form micropores. The emergence of microvoids may cause the material to soften, becoming a source of instability, and the shear band cannot be indefinitely delayed. Therefore, it is crucial to identify and apply suitable materials in appropriate service conditions in order to evaluate their high-strain-rate performance and prevent the occurrence of catastrophic failure.

## Figures and Tables

**Table 1:** Nomenclature and value of constants and variables used in Equation (1).

Constant and Variable	Meaning	Value
*L*	Average subgrain diameter	-
*k*	Boltzmann’s constant	1.38 × 10^−23^ J·K^−1^
*T*	Absolute temperature	-
*δ*	Grain boundary thickness	-
*η*	Grain boundary energy	-
*D*_bo_	Constant related to grain boundary diffusion	-
*Q*	Activation energy for grain boundary diffusion	-
*θ*	Subgrain misorientation	0–30°
*R*	Gas constant	8.314 J·mol^−1^·K^−1^

**Table 2:** Summary of grain refinement and temperature estimation in the ASB of various materials.

Materials	Initial Grain Size	Grain Size in ASB	Estimated Temperature	Reference
Pure Ti	20 μm	0.1–1 μm	930 K (0.48 Tm)	[117]
Ultrafine-grained pure Ti	120 nm	40 nm	900 K (0.46 Tm)	[145]
CrMnFeCoNi high-entropy alloy	8 μm	100–300 nm	700 K (0.40 Tm)	[62]
Pure Ti	80 μm	6 μm	1400–1600 K (0.72–0.82 Tm)	[154]
Ti-5Al-5Mo-5V-1Cr-1Fe alloy	-	50–200 nm	1132 K (0.62 Tm)	[115]
Ti-6Cr-5Mo-5V-4Al alloy	150 μm	100–300 nm	873–967 K (0.45–0.50 Tm)	[155]
Pure zirconium (Zircadine 702)	7.5 μm	200 nm	930 K (0.43 Tm)	[144]
Ti-5Mo-5V-8Cr-3Al alloy	-	250 nm	940 K (0.50 Tm)	[156]
Pure Ti	24 μm	0.1–1 μm	900 K (0.46 Tm)	[157]
AZ31 alloy	35 μm	100 nm	-	[158]
Al0.1CoCrFeNi high-entropy alloy	500 μm	300 nm	473 K (Limited temperature rise)	[138]
90W-7Ni-3Fe alloy	30 μm	60–200 nm	Limited temperature rise	[28]
Ti-10V-2Fe-3Al alloy	327 μm	174 nm	673 K (0.38 Tm)	[11]

## Data Availability

The raw/processed data required to reproduce these findings cannot be shared at this time as the data also form part of an ongoing study.
